# Single-shot readout of a superconducting qubit using a thermal detector

**DOI:** 10.1038/s41928-024-01147-7

**Published:** 2024-04-10

**Authors:** András M. Gunyhó, Suman Kundu, Jian Ma, Wei Liu, Sakari Niemelä, Giacomo Catto, Vasilii Vadimov, Visa Vesterinen, Priyank Singh, Qiming Chen, Mikko Möttönen

**Affiliations:** 1 QCD Labs, QTF Centre of Excellence, Department of Applied Physics, Aalto University, Aalto, Finland; 2 IQM, Espoo, Finland; 3 QTF Centre of Excellence, VTT Technical Research Centre of Finland Ltd, Espoo, Finland

**Keywords:** Superconducting devices, Qubits, Electrical and electronic engineering, Superconducting devices, Electronic devices

## Abstract

Measuring the state of a qubit is a key fundamental operation of a quantum computer. High-fidelity single-shot readout of superconducting qubits can be achieved using parametric amplifiers at millikelvin temperatures. However, scaling parametric amplifiers beyond hundreds of qubits is challenging due to practical size and power limitations. Nanobolometers can, in contrast, offer scalability, sensitivity and speed suitable for qubit readout. Here we show that a bolometer can provide single-shot qubit readout with a readout duration of 13.9 μs and a single-shot fidelity of 0.618. The fidelity is mainly limited by the energy relaxation time of the qubit (28 μs), and a fidelity of 0.927 is found after removing errors arising from this relaxation. In the future, higher-fidelity single-shot readout may be achieved through improvements in chip design and experimental setup, as well as a change in the bolometer absorber material to reduce the readout time to the level of hundreds of nanoseconds and below.

## Abstract (web summary)

Bolometers can be used for qubit readout, offering a scalable, sensitive and high-speed alternative to current parametric-amplifier-based approaches.

## Main

Qubit readout in a quantum computer is required to determine the result at the end of a computation^1,2^ as well as for error correction, which is necessary for fault tolerance^3–5^. Superconducting qubits are a promising platform for practical quantum computing^6–8^, and readout is one of the main bottlenecks restricting the development of quantum-error-corrected large-scale superconducting quantum processors. In particular, recent experiments have shown that the readout phase is a key source of error in error correction cycles^9–11^.

The most common method of measuring the state of superconducting qubits in the framework of circuit quantum electrodynamics is known as dispersive readout^6,12,13^. Here a qubit is off-resonantly coupled to a readout resonator, the frequency of which shifts depending on the qubit state. With this approach, single-shot readout fidelity above 99% with an averaging time below 100 ns has been achieved for single qubits^14,15^, and 97–99% on average for the simultaneous multiplexed readout of several qubits^10,11,16^.

To achieve a signal-to-noise ratio (SNR) sufficient for high-fidelity single-shot dispersive readout, the output signal from the readout resonator is typically amplified at the millikelvin stage by a parametric amplifier, such as a Josephson parametric amplifier^17^ or a travelling-wave parametric amplifier (TWPA)^18^. These amplifiers can be quantum limited, where the noise added by the amplification stems solely from the Heisenberg uncertainty principle for the in-phase and quadrature components of the amplified signal^19–22^. Parametric amplifiers can offer high gain and low noise, but introduce challenges in terms of scaling to large numbers of qubits. These challenges include narrow bandwidth, which is undesirable in multiplexed qubit readout^16^. Although TWPAs provide a broad bandwidth, they typically incorporate more than 10^3^ Josephson junctions, making their on-chip footprint large and high-yield large-scale fabrication challenging. Importantly, both types of amplifier currently require strong isolation between the amplifier and qubit–resonator system: Josephson parametric amplifiers work in the reflection mode via a circulator, and TWPAs utilize a relatively strong pumping tone potentially near the qubit or resonator frequencies. TWPAs also amplify vacuum and other noise, which may reflect back into the TWPA input and cause decoherence in the qubit. To mitigate these issues, permanent-magnet-based microwave isolators are typically placed between the readout transmission line and amplifier input. Such isolators introduce losses in the signal. They are also large, costly and require shielding to protect both qubit and amplifier from the magnetic fields they introduce.

These scalability issues have driven the development of alternative readout techniques. Microwave photon counters offer an alternative to voltage amplification at the millikelvin stage, but require a more involved pulse sequence and suffer from backaction arising from the creation of quasiparticles and their tunnelling. Qubit readout can also be achieved without a parametric amplifier by driving the qubit to the second excited state before readout^23^. However, the required non-parametric microwave amplifier introduces high-temperature noise close to the qubit frequency, which necessitates bulky microwave isolators. Another approach to promote scalability is to deliver signals to and from the cryostat in the optical domain^24,25^.

Nanobolometers^26–28^ have been shown to be fast and sensitive enough for the readout of superconducting qubits, reaching thermal time constants in the range of hundreds of nanoseconds and energy resolution of a few typical microwave photons^29^. Such bolometers have several attractive properties for qubit readout. In contrast to parametric amplifiers, bolometers can be driven by a sub-gigahertz probe tone^26^, a frequency well below the typical qubit and resonator frequencies of 4–8 GHz. In addition, bolometers can be probed with a low power of approximately −120 or −130 dBm at the chip^27^, whereas TWPAs typically require pump powers of −75 dBm or higher^18^. Furthermore, the absorbing port of the bolometer can be conveniently matched to 50 Ω (ref. ^28^). The absorber presents a cold bath to the readout transmission line, allowing for the detection frequency to vary by orders of magnitude given a fixed probe frequency^30^. These features may remove the need for isolators between the qubit–resonator system and the bolometer; together with the small size of the bolometers, this makes them promising in terms of scalability.

Since a bolometer measures power, or photon number, it is not bound to add quantum noise stemming from the Heisenberg uncertainty principle. The vacuum noise does not promote detection events in the bolometer since no energy can be extracted from a vacuum. Thus, bolometric readout is fundamentally different from the readout utilizing voltage amplification. In addition, bolometers are relatively simple to fabricate and operate^28^. They do not require engineering of many small Josephson junctions, and a bolometer requires only a single continuous probe tone with two parameters—power and frequency—to optimize performance.

In this Article, we report the integration of a sensitive bolometer at millikelvin temperatures with the readout circuitry of a superconducting qubit. We demonstrate single-shot qubit readout at a fidelity of 0.618. Our experiment does not use the fastest compatible bolometers^29^, but those that are orders of magnitude slower^28^, and thus, bolometric single-shot qubit readout has great potential for the high-fidelity scalable readout of superconducting qubits.

## Qubit-to-bolometer setup

**Fig. 1:**
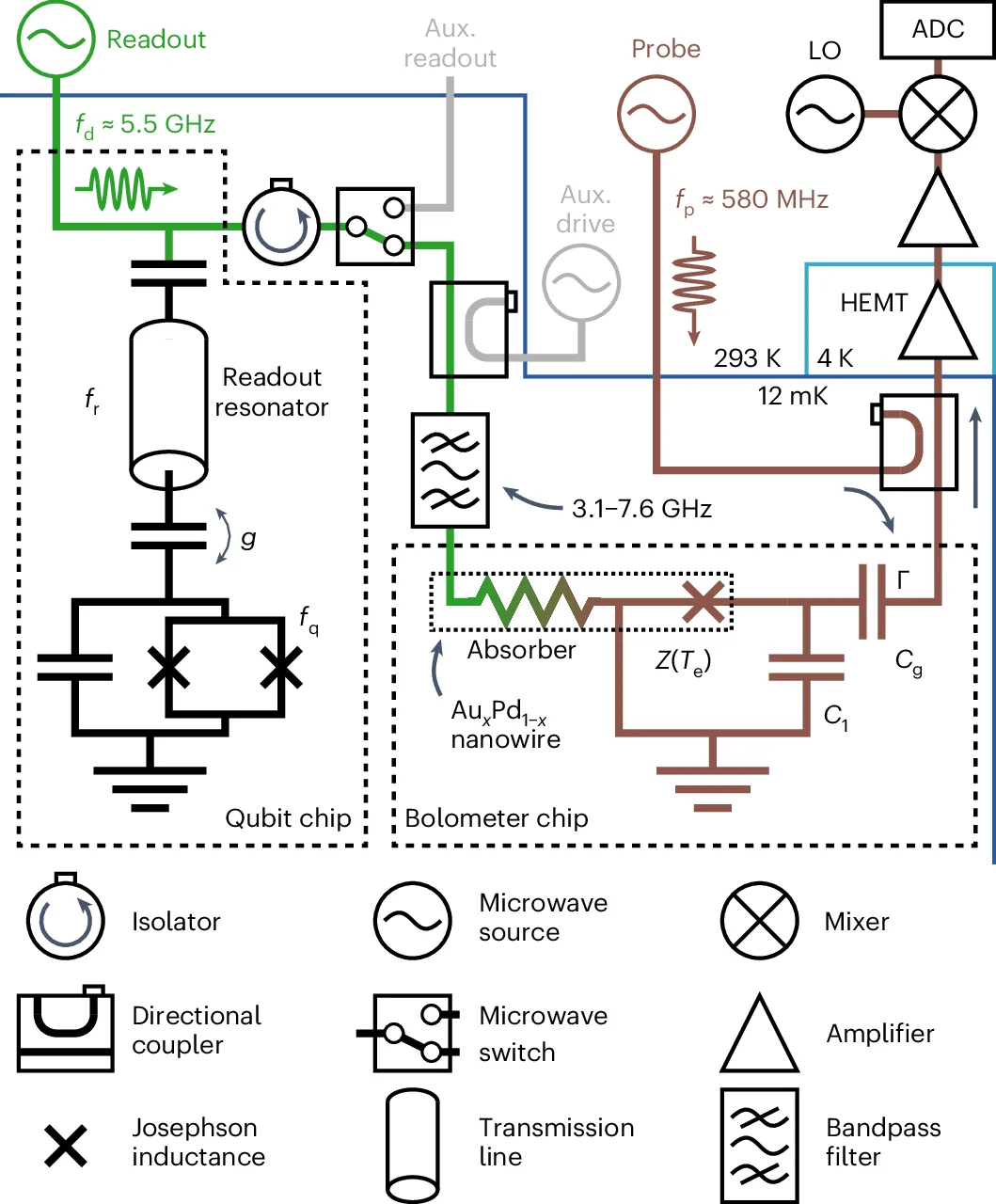
Experimental setup. The readout resonator is driven at a frequency of *f*_d_ ≈ 5.5 GHz by a readout tone, which is reflected off of the resonator and absorbed by a metallic nanowire in the absorber port of the bolometer. The absorbed radiation shifts the electron temperature *T*_e_ and, consequently, the impedance *Z*(*T*_e_) of a chain of SNS junctions, marked by a brown cross. A continuous probe tone with a frequency of *f*_p_ ≈ 580 MHz is reflected off of the gate capacitor *C*_g_ of the bolometer (as determined by reflection coefficient *Γ*). The reflected signal is amplified, digitized and used to determine the qubit state. The qubit can be prepared in its excited state by driving through an additional qubit drive line, which is not shown in this schematic (Methods and Extended Data Fig. 1). Aux., auxiliary; ADC, analogue-to-digital converter; LO, local oscillator.

**Extended Data Fig. 1:**
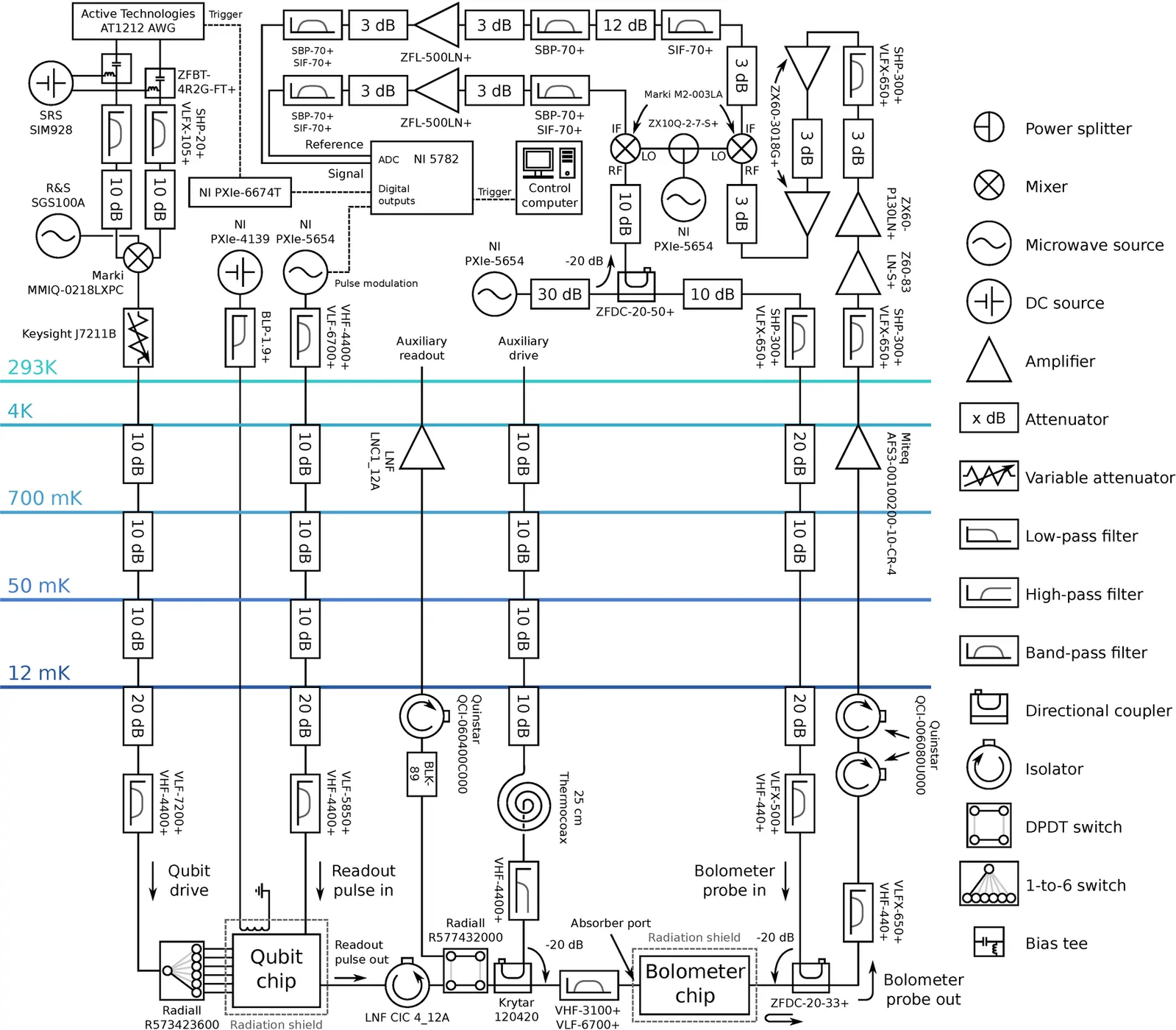
Full experimental setup. The labels for the filters, directional couplers, amplifiers, and the power splitter refer to mini-circuits model numbers. The band-pass filters with two labels denote a low-pass filter and high-pass filter in series.

**Extended Data Fig. 2:**
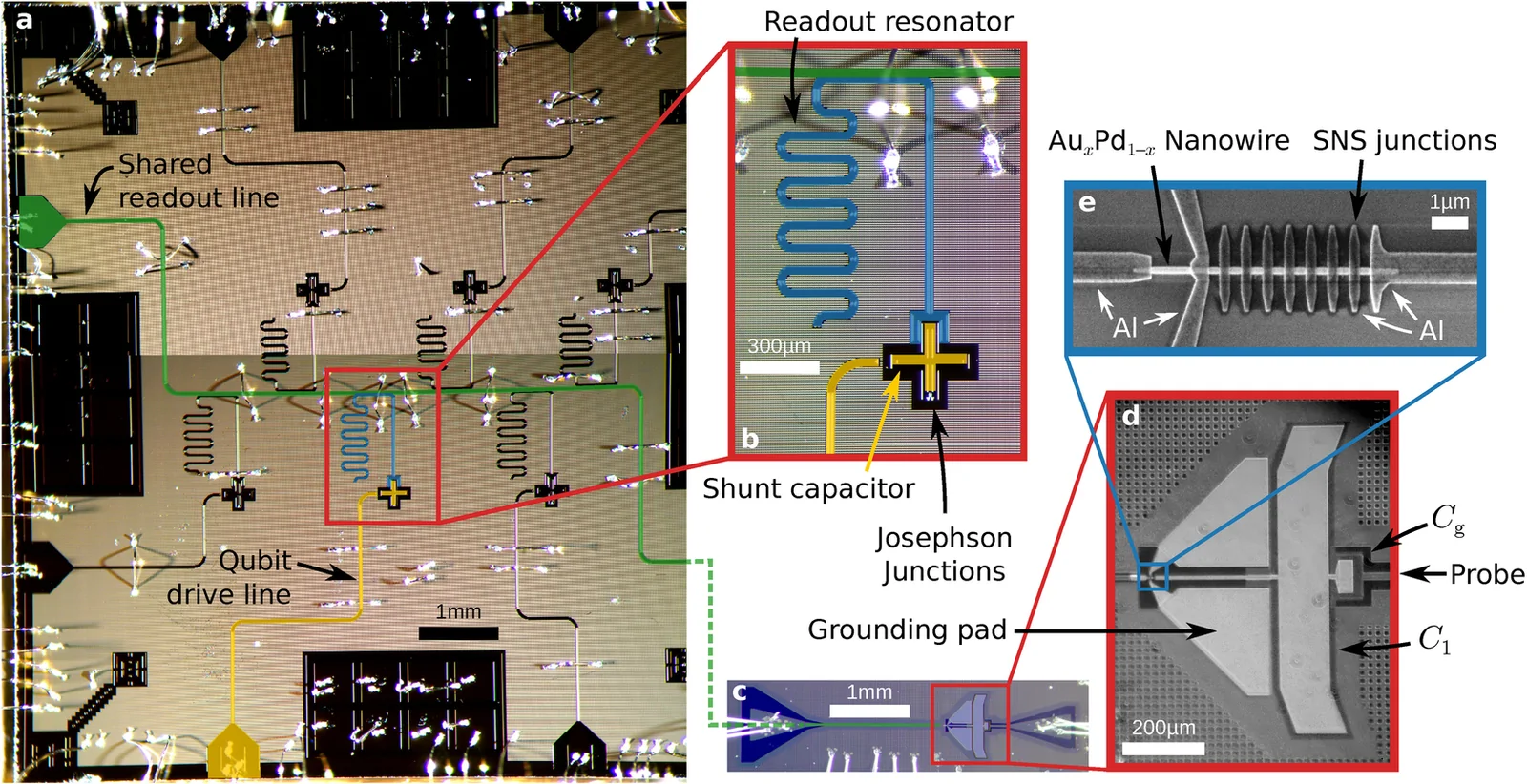
Chip design. (a) False-color optical micrograph of the qubit chip composited from two images. The chip contains six qubits with individual drive lines and readout resonators, with a shared readout line (highlighted in green). (b) Enlarged view of one of the qubits, showing the readout resonator (blue), the drive line (yellow), and the cross-shaped qubit capacitor (yellow). The Josephson junctions are near the bottom of the capacitor. (c) False-color optical micrograph of the bolometer chip, to scale with (a). The readout line from the qubit chip is connected to the transmission line (green) leading to the absorber of the bolometer. (d) Area enclosed in the red rectangle in (c) showing the Al grounding pad, bolometer shunt capacitor *C*_1_, and the probe gate capacitor *C*_g_. The circular artifacts originate from dust particles on the microscope lens. (e) Scanning-electron micrograph of the AuPd nanowire and Al islands forming the superconductor–normal-metal–superconductor (SNS) junctions of a bolometer similar to that used in this paper.

A schematic of the experimental setup is shown in Fig. 1 (Extended Data Fig. 1 provides further details). The qubit–resonator system and bolometer reside on separate chips (Extended Data Fig. 2). The qubit chip consists of six typical flux-tunable Xmon qubits^31^, each with a capacitively coupled coplanar waveguide readout resonator in a notch configuration. The readout resonators share a common readout feedline and the flux is adjusted using a shared external magnetic coil; otherwise, the qubits are uncoupled and individually addressable via separate qubit drive lines (Methods). The qubit–resonator pairs have varying qubit and resonator frequencies, as well as qubit–resonator coupling strengths.

We choose a qubit with a transition frequency of *f*_q_ = 7.655 GHz at the flux sweet spot, anharmonicity *α*/(2π) = −273 MHz, energy relaxation time of *T*_1_ = 28.0 μs and Ramsey dephasing time of *T*_2_ = 7.6 μs. This qubit is coupled to a readout resonator with a coupling strength of *g*/(2π) = 61 MHz. The resonator has a fundamental resonance frequency of *f*_r,g_ = 5.473 GHz if the qubit is in its ground state |*g*〉 and linewidth of *κ*_r_/(2π) = 1.0 MHz. These parameters produce a dispersive shift of *χ*/2π = −0.3 MHz, that is, the resonator frequency shifts to *f*_r,e_ = *f*_r,g_ + 2*χ*/(2π) if the qubit is in its excited state |*e*〉.

To read the qubit state, a rectangular microwave pulse of length *t*_RO_ ≈ 10 μs, frequency *f*_d_ and power *P*_d_ is applied to the feedline of the readout resonator. In a typical dispersive readout, the drive frequency is chosen to be in the middle of the dressed resonator frequencies, that is, *f*_d_ = (*f*_r,g_ + *f*_r,e_)/2, and the photons reflected from the resonator accumulate a phase shift depending on the qubit state^6^. In contrast, here we operate in the photodetection mode^32^ by driving close to one of the dressed frequencies, namely, *f*_d_ ≈ *f*_r,g_. In this driving scheme, information about the qubit state is mostly carried by the power of the signal, that is, the number of photons emitted by the resonator into the feedline. A power difference is necessary for the bolometer, since it is a thermal detector, sensitive to the power but insensitive to the phase of its input signal. Note that for typical dispersive readout, the ratio ∣*χ*∣/*κ*_r_ = 1/2 yields the optimal SNR^33^, whereas for photodetection-based readout, the optimal ratio is higher^32^.

The output of the feedline for the readout resonator is connected to the absorber port of the bolometer chip, which is similar to the device discussed elsewhere^28^. The main component of the bolometer is a resistive Au_*x*_Pd_1−*x*_ (*x* ≈ 0.6) nanowire. A segment of the nanowire works as the resistive absorber and the remaining wire is interrupted by a series of superconducting Al islands, forming a chain of superconductor–normal metal–superconductor (SNS) junctions. The impedance *Z*(*T*_e_) of this junction chain depends on the electron temperature of the nanowire, *T*_e_. The junction chain is embedded in an effective *LC* circuit formed by a shunt capacitor of *C*_1_ = 134 pF in parallel with *Z*(*T*_e_), which can be modelled as a parallel resistance and inductance^27^. The nanowire is grounded between the absorber and junctions, so that the essentially purely real-valued impedance of the absorber does not contribute to the *LC* circuit.

The bolometer is probed by reflecting a continuous tone at power *P*_p_ and frequency *f*_p_ from the gate capacitor *C*_g_ = 0.87 pF. With a low *P*_p_ value, the *LC* circuit resonates at a frequency of *f*_b_ = 585 MHz with a linewidth of 7.6 MHz. As radiation is absorbed by the absorber, *T*_e_ increases, which shifts *f*_b_ down, and thus, by observing changes in the reflection coefficient *Γ* at the gate capacitor, it is possible to detect the radiation incident on the bolometer input. The reflected probe signal is amplified by a low-noise high-electron-mobility-transistor (HEMT) amplifier at 4 K, and further amplified, demodulated and digitized at room temperature in a heterodyne configuration.

**Fig. 2:**
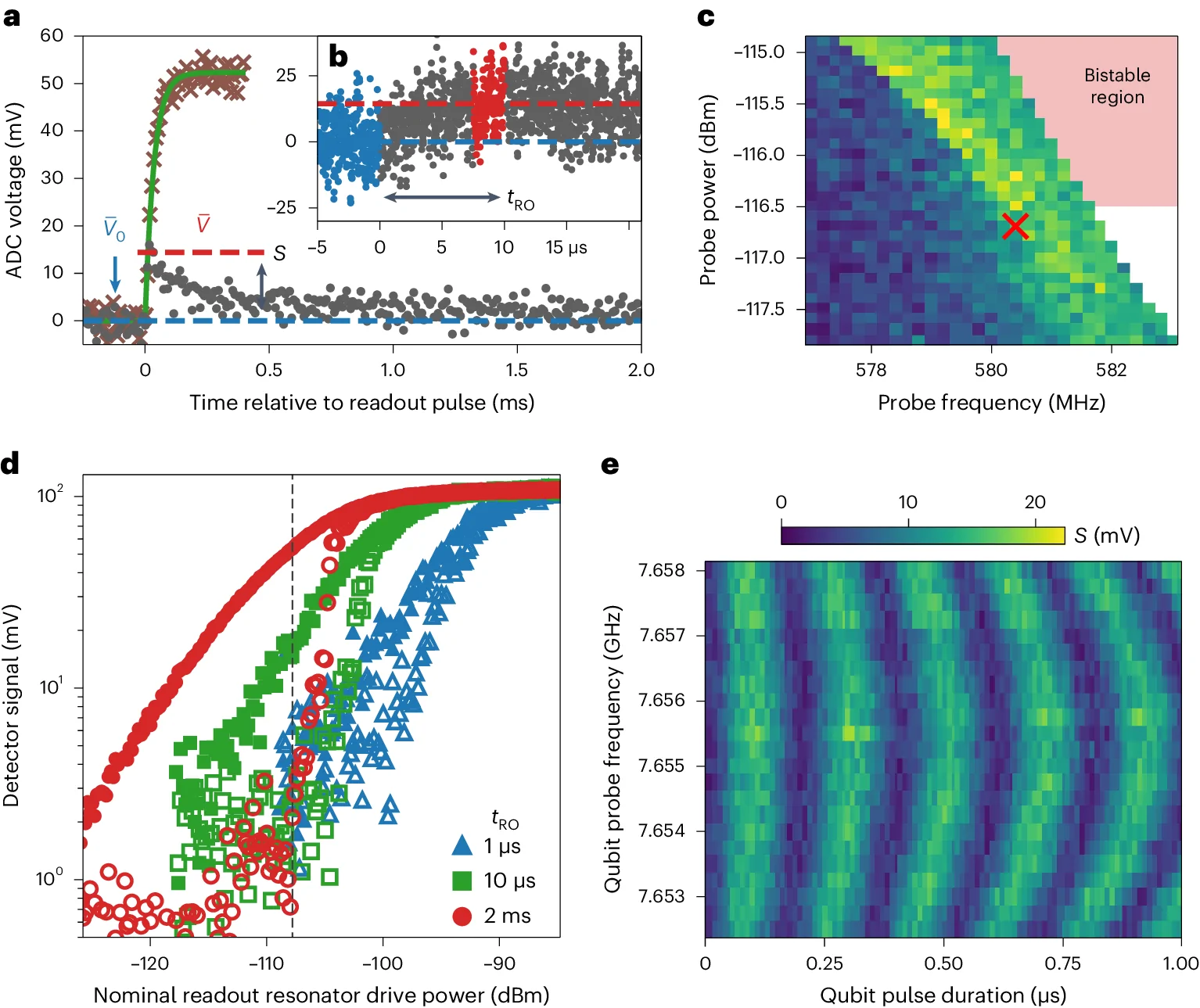
Characterization experiments. **a**, Example time trace of the probe signal reflected from the bolometer, with a qubit readout pulse length of *t*_RO_ = 10 μs (grey dots) and >1 ms (brown crosses). The solid green line is an exponentially rising fit to the long pulse, and the dashed horizontal lines indicate the extracted values of $\bar{V}$ and ${\bar{V}}_{0}$ used to define the detector signal *S* for the short pulse. The parameter values in all the panels are shown in Table 1, unless specified otherwise. **b**, Similar data as in **a**, but only for the 10 µs readout pulse. The blue- and red-coloured regions indicate where ${\bar{V}}_{0}$ and $\bar{V}$ are averaged, respectively. These data are taken with 128 ensemble averages. The signal appears noisier than that in **a**, since each data point in **a** is calculated by averaging 512 adjacent data points. **c**, Detector signal *S* as a function of probe frequency *f*_p_ and probe power *P*_p_. In the region shaded with red, where *P*_p_ ≳ −116.5 dBm, the bolometer exhibits bistability due to electrothermal feedback. The red cross indicates the chosen operation point for qubit readout. The same colour bars are also used in **e**. **d**, Detector signal as a function of the nominal power of readout pulse *P*_d_ for various indicated readout pulse lengths and for the readout pulse applied off resonance (*f*_d_ = 5.400 GHz, filled markers) and on resonance (*f*_d_ = 5.473 GHz = *f*_r,g_, unfilled markers). The resonator becomes considerably nonlinear at approximately −108 dBm, indicated by the black dotted vertical line. **e**, Detector signal as a function of the resonant qubit drive pulse length and frequency, showing Rabi oscillation. These data are taken with 512 ensemble averages.

**Extended Data Fig. 3:**
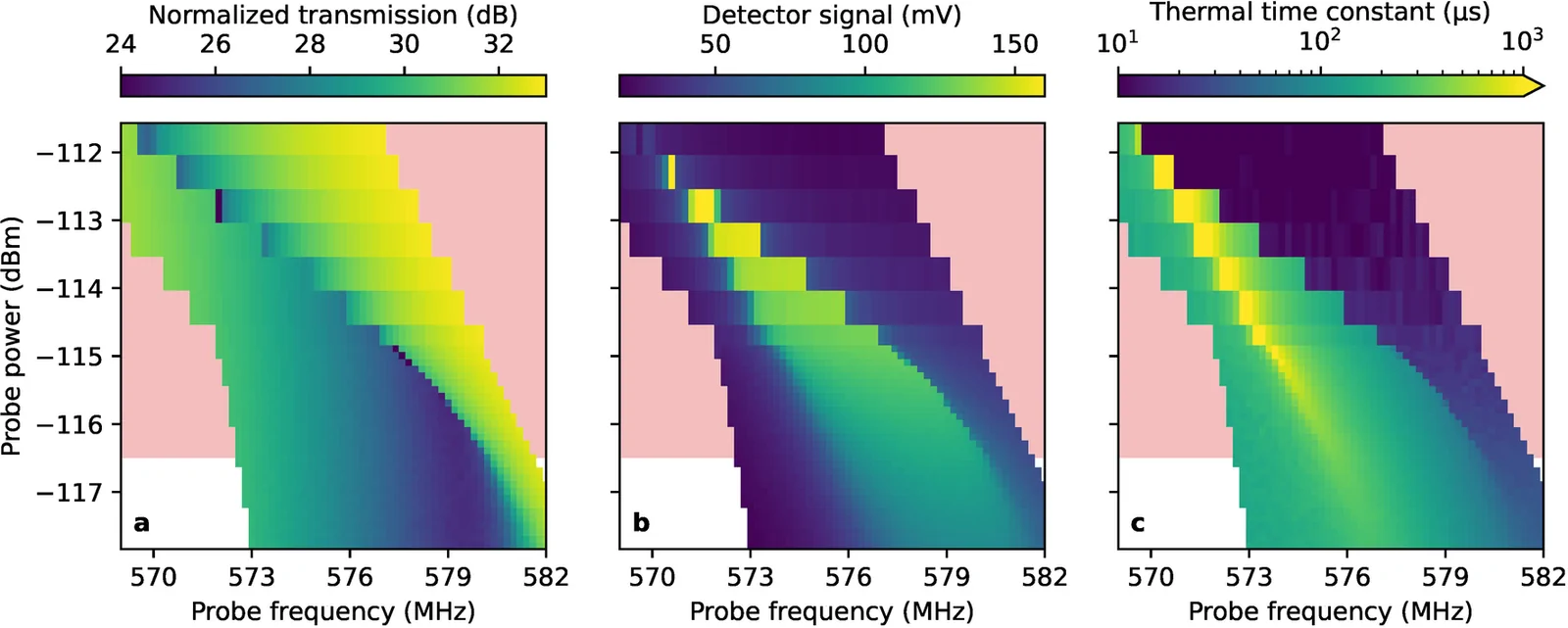
Bolometer characterization. (a) Total transmission power divided by the reference power, (b) detector signal *S*, and (c) extracted thermal time constant *τ*_b_ of the bolometer as functions of the probe frequency *f*_p_ and power *P*_p_. The red background approximately indicates the power region where the bolometer exhibits bistability.

The shift in *f*_b_ due to the readout pulse coming from the readout resonator is observed as a change in the digitized voltage (Fig. 2a,b). For low *P*_p_, the readout pulse causes *T*_e_, and thus, the reflected signal to exponentially approach a steady-state value with a thermal time constant *τ*_b_. The time constant depends on *f*_p_, *P*_p_ and the power of the readout pulse *P*_d_ in a non-trivial way due to electrothermal feedback^27,28^. For the relevant parameter regime considered here, *τ*_b_ varies between 10 μs and 1 ms (Extended Data Fig. 3c). In particular, we have *τ*_b_ ≳ *T*_1_, which implies that for qubit readout, we must operate the bolometer in a calorimetric fashion, that is, with *t*_RO_ < *τ*_b_. This is highlighted in Fig. 2a, where the time constant, extracted from a measurement with a long readout pulse of *t*_RO_ > 1 ms, is *τ*_b_ = 36.2 μs. With *t*_RO_ = 10 μs, which is more feasible for qubit readout than *t*_RO_ > *τ*_b_, the steady state is far from the reached maximum signal level.

For high-enough *P*_p_, the electrothermal feedback results in bistability for the electron temperature of the SNS junction. This bistability can be exploited for high-fidelity photodetection by operating the bolometer in the latching mode using a pulsed probe tone^27^. However, this scheme introduces substantial dead time for the detection, and requires detailed calibration of the pulse shape. For simplicity, we, thus, focus on the continuous detection scheme with a sufficiently low *P*_p_ value.

Using the time-domain data digitized from the bolometer output, we define the detector signal as

1 $$\begin{array}{r}S=\bar{V}-{\bar{V}}_{0},\end{array}$$

where ${\bar{V}}_{0}$ is the time average of the digitized voltage before the readout pulse and $\bar{V}$ is the time average of the voltage over some averaging window [*t*_0_, *t*_RO_]. Since *t*_RO_ < *τ*_b_, increasing the time over which the average is taken can substantially decrease $\bar{V}$, but may increase the SNR. In practice, we observe that choosing *t*_0_ = 0.75 × *t*_RO_ balances these two effects reasonably well.

Both qubit–resonator and bolometer chips are mounted on their individual sample holders, which are placed in separate magnetic shields and attached to the mixing-chamber plate of a dilution refrigerator with a base temperature of 12 mK. Note that in the setup presented in Fig. 1, an additional microwave switch and directional coupler are placed between the qubit and bolometer. These allow measuring the qubit and driving the bolometer individually, and are only used for the initial separate characterization of the qubit and bolometer. The auxiliary readout channel called for the use of an isolator between the chips, but this can be removed in future experiments.

## Qubit measurements

During the initial characterization, we apply no driving to the readout resonator of the qubit and find the bolometer resonance by measuring the reflection coefficient of the bolometer probe signal as a function of *f*_p_ and *P*_p_. Next, we apply pulses to the readout resonator with *f*_d_ well detuned from *f*_r,g_, and map the detector signal *S* as a function of *f*_p_ and *P*_p_. These results (Fig. 2c and Extended Data Fig. 3) provide us with a feasible operation point (*f*_p_, *P*_p_) = (580.5 MHz, −116.7 dBm) where *S* is maximized with *P*_p_ below the region where the electrothermal feedback induces bistability, as discussed above. Table 1 summarizes the parameter values used during the initial characterization.

**Table 1:** Typical parameter values used during characterization measurements

Quantity	Symbol	Value
Bolometer probe frequency	*f*_p_	580.5 MHz
Bolometer probe power (nominal)	*P*_p_	−116.7 dBm
Readout resonator drive frequency	*f*_d_	5.400 GHz or *f*_r,g_
Readout drive power (nominal)	*P*_d_	−107.8 dBm
Readout pulse length	*t*_RO_	10 µs
Number of ensemble averages		16

Note that all the power values reported in this Article are uncalibrated, and the nominal values reaching the corresponding chip are based on the estimated attenuation of the lines in our setup.

With the bolometer operation point fixed, we carry out common qubit characterization measurements^34^ and monitor the bolometer signal. First, we use single-tone spectroscopy to find the resonator frequency (Extended Data Fig. 6a), and confirm that it matches the results obtained from the initial characterization carried out via the auxiliary readout port. We also measure *S* as a function of the qubit readout power *P*_d_, both on resonance with *f*_d_ = *f*_r,g_ and off resonance with *f*_d_ = *f*_r,g_ − 70 MHz, with no excitation pulse applied to the qubit. These results are shown in Fig. 2d for different readout pulse durations *t*_RO_. The difference between the on-resonance and off-resonance values of *S* corresponds to the maximum contrast attainable with this readout scheme if the dispersive shift *χ* of the qubit–resonator system was infinite. Importantly, we observe that the suitable values of *P*_d_ for qubit readout are near the minimum power the bolometer can detect in the given integration time. The highest feasible readout power is *P*_d_ ≈ −108 dBm, above which the on-resonance signal exhibits a sudden jump since the resonator becomes nonlinear and the quantum non-demolition (QND) nature of the readout breaks down^35,36^. With a very long *t*_RO_ of 2 ms, a good signal is achieved for *P*_d_ well below the onset of nonlinearity. However, reducing *t*_RO_ to 1 μs, which is still an order of magnitude longer than current state-of-the-art qubit readout, a reasonable SNR is not achieved even with *P*_d_ ≈ −108 dBm. The low SNR with short *t*_RO_ further highlights that the long bolometer time constant limits the readout signal. Fortunately, a graphene-based bolometer^29^ seems suitable for operation at the 100 ns timescale.

After characterizing the resonator, we find an estimate for the qubit frequency using two-tone spectroscopy (Extended Data Fig. 6b). Figure 2e shows the subsequent results of a Rabi oscillation measurement where we initialize the qubit to its ground state, drive it with a rectangular pulse of variable length and frequency, and measure the readout resonator using the bolometer. We observe a clear chevron pattern as desired, from which we determine a π-pulse length of 100 ns. Here we employ a 10 μs readout pulse and ensemble average the result 512 times. Importantly, we do not observe any drift in the bolometer signal as a function of the pulse length and the contrast between the local minima and maxima in Fig. 2e does not noticeably deteriorate even with a 1 µs Rabi length, indicating that the qubit drive pulse is not leaking to the bolometer.

**Fig. 3:**
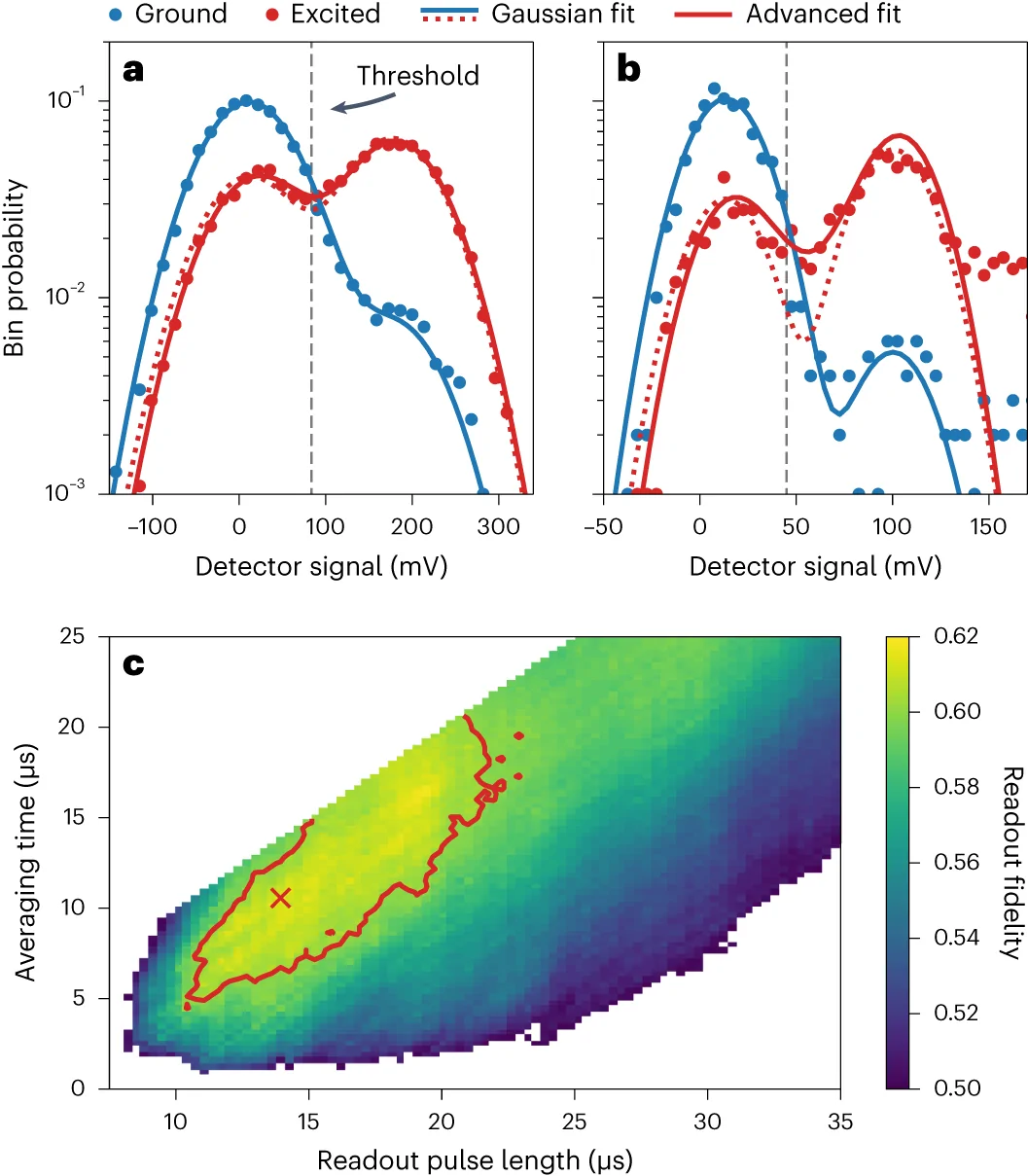
Single-shot readout. **a**, Probability distributions of the single-shot qubit readout signal *S* for the qubit prepared in the ground (blue dots) and excited (red dots) states. The solid blue line and dotted red line are fits to a sum of two Gaussian distributions, whereas the solid red line is a fit to the excited-state data using a model that accounts for the *T*_1_ decay in the qubit during readout (Methods). The vertical dashed line indicates the threshold that yields the highest readout fidelity. The bolometer is tuned to its operation point and the readout power is *P*_d_ = −108 dBm, with a pulse length of *t*_RO_ = 20 μs and an averaging time of 5 μs for $\bar{V}$. **b**, Similar data as in **a**, but for *t*_RO_ = 13.9 μs and an averaging time of 10.6 µs for $\bar{V}$ and long averaging for ${\bar{V}}_{0}$ (see the main text). **c**, Single-shot readout fidelity as a function of the readout pulse length and averaging time for $\bar{V}$, both changed during post-processing. The red boundary indicates the region where the fidelity is greater than 0.6. The highest fidelity is achieved with the parameters indicated by the red cross, where the data in **b** are obtained.

With the qubit frequency and π-pulse length calibrated, we carry out single-shot qubit readout by alternating between preparing the qubit in |*g*〉 and |*e*〉, and recording *S* with no ensemble averaging. For each prepared state, we record 10^4^ data points. The data are binned to produce a histogram, with the bin width chosen using Scott’s rule^37^. To maximize the readout fidelity, defined as *F* = 1 − *P*(*g*∣*e*) − *P*(*e*∣*g*), we optimize the threshold value for *S* to decide the measurement outcome of |*g*〉 or |*e*〉. Here *P*(*a*∣*b*) is the probability of measuring the qubit in state |*a*〉 provided that it was prepared in state |*b*〉, where the probabilities are obtained from the measured distributions. The highest fidelity is obtained by increasing the readout pulse length to *t*_RO_ = 20 μs, so that the above-discussed choice of *t*_0_ = 0.75 × *t*_RO_ yields an averaging time of 5 μs for $\bar{V}$, and setting the readout power to *P*_d_ = −108 dBm, which is just below the point of nonlinearity for the readout resonator. Figure 3a shows the measured probability distributions for the probe signal *S* with these parameters, from which we extract the fidelity as *F* = 0.49.

In an effort to further optimize the single-shot readout fidelity, we carry out an additional experiment, where we intentionally stretch the readout pulse to be unreasonably long (that is, 40 μs) and instead of just storing *S* derived from the time-averaged quantities ${\bar{V}}_{0}$ and $\bar{V}$, we record the full time traces of the bolometer output signal for 1,000 single shots. With these data, we may vary the effective readout pulse length and averaging time of $\bar{V}$ during post-processing. Figure 3c shows the resulting single-shot readout fidelity as a function of the digitally determined pulse length and averaging time. We observe a region of relatively high fidelity (*F* > 0.600; Fig. 3c (red boundary)), with the highest fidelity of 0.618 achieved with *t*_RO_ = 13.9 μs and *t*_RO_ − *t*_0_ = 10.6 μs. Figure 3b shows the probability distributions of the signal with these parameter values.

The fidelity is overall higher in Fig. 3b,c than in Fig. 3a. This is mostly because in the latter, ${\bar{V}}_{0}$ is individually calculated for each single shot. Since each value of ${\bar{V}}_{0}$ is averaged over a relatively short 1.1 µs window, it introduces considerable noise into the value of *S* calculated using equation (1). In contrast, a common value of ${\bar{V}}_{0}$ is used for the whole dataset in Fig. 3b,c, meaning that only the fluctuations in $\bar{V}$ contribute to the noise in *S*. Rescaling the standard deviation of the data in Fig. 3b,c by a factor corresponding to the noise from this 1.1 µs window reproduces the increased noise level shown in Fig. 3a.

For the qubit prepared in |*e*〉, the single-shot probability distribution is clearly bimodal (Fig. 3a,b). This is expected, since *t*_RO_ is comparable with the qubit energy decay time *T*_1_, and thus, the qubit experiences considerable spontaneous relaxation during the readout. Even in the case of infinitely narrow distributions and ideal state preparation, the *T*_1_ decay produces an error of approximately 1 − exp(−*t*_RO_/(2*T*_1_)) ≈ 22% for *t*_RO_ = 13.9 μs and *T*_1_ = 28.0 μs (ref. ^38^). The long readout stems from the relatively long effective thermal time constant of the bolometer, namely, *τ*_b_ = 9.4 μs, extracted from the data in Fig. 3c for the qubit prepared in |*g*〉. In Fig. 3c, the trend of relatively high and constant fidelity obtained for simultaneously increasing readout pulse length and averaging time indicates that the increase in fidelity owing to the increasing bolometer SNR approximately compensates the decrease in fidelity owing to the increasing qubit decay. With short readout times, the fidelity is low because the distributions corresponding to the different qubit states are not well separated.

The data shown in Fig. 3a, as well as the |*g*〉-state data shown in Fig. 3b, are well modelled by a sum of two Gaussian distributions. This simple model deviates from the |*e*〉-state data shown in Fig. 3b between the centres of the Gaussian distributions, since it does not take into account the *T*_1_ decay of the qubit during readout. Fortunately, we capture this effect using an advanced model (Methods). For the |*e*〉-state data shown in Fig. 3a, there is no substantial difference between the Gaussian and advanced models, since the effect of decay on the distribution is masked by noise. Using the parameters extracted from these fits, we determine the fidelity with *t*_RO_ = 13.9 μs and the *T*_1_ error removed to be *F* ≈ 0.927 (Methods). The |*e*〉-state data shown in Fig. 3b deviate from the fit above roughly 150 mV. We attribute this to qubit excitations outside the computational subspace owing to the high readout power.

## Conclusions

We have shown that a thermal detector can achieve reasonable-fidelity single-shot readout of a superconducting qubit, measuring the power of the qubit readout signal, not its voltage. We reported qubit characterization measurements with less ensemble averaging than in previous setups lacking millikelvin amplifiers. Using a 13.9 µs readout pulse, we achieve a single-shot readout fidelity of 0.618, and a fidelity of 0.927 after removing errors arising from a finite qubit lifetime.

Several factors in our experiment can be improved to increase the single-shot readout fidelity. The SNR in the single-shot readout is fairly low, which mainly arises from the long effective thermal time constant of the bolometer—on the order of tens of microseconds. This requires the use of a long readout pulse, which degrades the fidelity due to *T*_1_ decay of the qubit. To reduce the time constant, a material with lower heat capacity than AuPd—such as graphene—can be used as the absorber^29,39^.

The long thermal time constant also introduces a long idle time between consecutive qubit measurements and prevents a demonstration of the QND nature of the bolometric readout. Fortunately, and regarding the qubit–resonator interactions, our scheme is essentially identical to the typical dispersive readout, the QND property of which is well known^6,14,15,19,38^. Minor modifications to our experimental setup may improve the SNR. In this work, we have a number of additional microwave components between the qubit and bolometer, which may be removed to reduce the signal loss between the two chips. Ultimately, the bolometer may be directly connected to the readout feedline, either on chip or in a layered flip-chip architecture^40^. Having the qubit and bolometer on separate chips is an important factor in degrading the fidelity in our experiment. By placing both devices on the same chip and optimizing the impedance matching between the readout resonator and bolometer absorber, undesired reflections can be eliminated (Methods and Extended Data Fig. 5).

The qubit chip design may also be optimized for photodetection-based readout. In our design, the readout resonator is placed in a notch-type configuration with symmetric coupling to the input and output feedlines. This is disadvantageous as half the signal reflected from the resonator is lost as it escapes through the input port. Changing to a transmission configuration with a weak coupling to the input port could substantially increase the power incident on the bolometer. The ratio ∣*χ*∣/*κ*_r_ = 0.3 in our sample is close to the value of 1/2, which maximizes the SNR in a typical dispersive readout, but it is sub-optimal for photodetection-based readout where a larger ratio is desirable. Furthermore, our readout resonator lies below the qubit frequency, which leads to the onset of non-adiabatic and chaotic resonator dynamics at a much lower photon number than for an elevated readout frequency^13,35,36^. Thus, designing the readout circuit to resonate well above the qubit frequency may lead to a greatly increased signal power at the bolometer input.

The readout fidelity can be improved by advanced pulsing schemes and improved data analysis methods. Instead of a simple rectangular pulse, the resonator can be driven by a two-step pulse^14^. This may enable the use of a power level that the bolometer can detect with a higher SNR, and still maintain the QND nature of the readout. Alternatively, the SNR of the signal reaching the bolometer may be increased by preparing the qubit in a highly excited state before readout^23^, or by using a two-tone drive that induces an effective longitudinal coupling between the qubit and bolometer^41,42^. Data analysis can be improved using optimized signal processing. For example, the time averaging can be weighted by the separation between the average trajectories corresponding to |*g*〉 and |*e*〉 (ref. ^14^), or the single-shot trajectories can be classified using a machine learning algorithm^43^. Here we have neglected the falling edge of the voltage signal that can be used in the advanced analysis methods to increase the SNR. The averaging over the falling edge does not increase the *T*_1_-related infidelity since during this averaging time as the readout pulse is off, and hence, the possible qubit decay will not lead to a change in the bolometer signal. Using a graphene bolometer in the calorimetric mode, which accounts for the falling edge, could improve the readout fidelity (Methods).

The next step for bolometric readout is to demonstrate its scalability. Placing qubits and bolometers on the same chip to perform high-fidelity readout without the need for isolators is yet to be experimentally demonstrated, to the best of our knowledge. Qubit readout should be multiplexed to minimize the number of necessary control lines. Multiple qubits can be read by coupling their readout resonators to individual bolometers and multiplexing the bolometers via a shared probe line. A chip containing three bolometers, which can probe in a frequency-multiplexed way, is being explored (Methods and Extended Data Fig. 7).

In large-scale quantum processors, it may be desirable to produce a binary output from the qubit readout signal inside the cryostat, thereby evading the need for room-temperature feedback. The bolometer can possibly be used for such a readout by operating in the bistable regime^27^, or by coupling the output to a click-type detector such as a Josephson bifurcation amplifier^44^. By incorporating the SNR improvements discussed above, we estimate that it may be possible to carry out single-shot qubit readout at 99.9% level of fidelity using bolometers (Methods). Thus, bolometers are a promising component for the scalable high-fidelity readout of superconducting qubits due to their low power consumption, large probe-tone frequency offset from qubits, resilience against quantum noise, small footprint, reduced need for microwave isolators and naturally introduced low-temperature bath for qubits that can be impedance matched at a broad range of readout frequencies.

## Methods

### Bolometer fabrication

To fabricate the bolometer, we begin with a four-inch silicon wafer (100) of high resistivity (*ρ* > 10 kΩ cm), covered by a 300 nm thermal oxide. Then, we sputter 200 nm of pure Nb onto the wafer. We use an AZ5214E photoresist in the positive mode with a hard contact to define the waveguide in a Karl Suss MA-6 mask aligner. After development, the sample undergoes etching using a Plasmalab 80Plus reactive ion etching system (Oxford Instruments). The plasma operates with a gas flow of SF_6_/O_2_ at 40/20 s.c.c.m. with a radio-frequency power of 100 W.

We clean the resist residuals in acetone and isopropyl alcohol using ultrasonic excitation and then dry the chip with a nitrogen gun. Next, we use an atomic layer deposition method to grow a 45 nm dielectric layer of Al_2_O_3_ in a Beneq TFS-500 system. Subsequently, we protect the dielectric layer at the desired capacitor regions using the AZ5214E resist, and wet etch the rest of the atomic-layer-deposited oxide with an ammonium fluoride–hydrofluoric acid mixture. We then cleave the four-inch wafer into a 2 × 2 cm^2^ chip using Disco DADdy.

The nanowire is patterned using EPBG5000pES electron-beam lithography system employing a bilayer of methyl methacrylate/polymethyl methacrylate resist on a single chip. We deposit a 30-nm-thick AuPd layer in an electron-beam evaporator at a rate of 0.5 Å s^−1^. After liftoff in acetone overnight, we galvanically pattern the superconducting leads connected to the nanowire by electron-beam lithography and deposit them with 100 nm of Al at a rate of 5 Å s^−1^. Finally, we cleave each pixel (5 × 5 mm^2^) using a laser micromachining system and package the chosen chip. We employ Al bonding wires to connect the chip to the printed circuit board of the sample holder. Extended Data Fig. 2c–e shows the micrographs of the bolometer chip.

### Qubit fabrication

The qubit samples are fabricated using the following steps. First, a layer of 200 nm of Nb is sputtered onto a high-resistivity silicon substrate. Next, we define the transmission line, readout resonator and transmon shunt capacitor using photolithography, followed by dry reactive ion etching.

The Al/Al_2_O_3_/Al Josephson junctions are subsequently fabricated using electron-beam lithography and the Dolan bridge method, where oxidation is used between the deposition of two Al layers to form the Josephson junctions. To ensure a galvanic contact between Al and Nb, the niobium oxide is removed by argon milling before the deposition of any Al.

The excess metal is lifted off in acetone. The room-temperature resistance of the Al/Al_2_O_3_/Al junctions is measured to select a sample that will most probably yield the desired qubit frequency. Finally, the chip is diced into individual samples and the selected sample is wire bonded to a sample holder. Extended Data Fig. 2a,b shows the micrographs of the qubit chip.

### Details of the experimental setup

Extended Data Fig. 1 shows a diagram of the full experimental setup. A PXI control computer initiates a measurement by sending a software trigger to a National Instruments NI-5782 transceiver module connected to an NI PXIe-7962R analogue-to-digital converter running custom field-programmable gate array code. The transceiver sends further digital trigger signals to initiate pulses to the readout resonator as well as excite the qubit. The clocks of all the devices are synchronized using a 10 MHz Rb reference.

The qubit excitation pulse is generated by an Active Technologies AT1212 digital-to-analogue converter, upconverted to the gigahertz range and directed into the cryostat via a series of attenuators and filters. A microwave switch placed at the mixing-chamber plate is used to select which of the six qubits on the chip is being driven. The qubit frequency is adjusted by applying a magnetic field to the qubit–resonator chip using a hand-wound coil outside the sample holder.

The readout pulse is generated by a microwave source using pulse modulation. The pulse is reflected off of the resonator on the qubit–resonator chip, after which it is directed through an isolator, a double-pole double-throw switch, a directional coupler, a high-pass filter with a 3 dB cutoff frequency at 3.1 GHz and a low-pass filter with a 3 dB cutoff frequency at 7.6 GHz, before reaching the bolometer absorber port. We have chosen the filters in an effort to place the passband around the readout frequency and filtering out possible leakage from the qubit drive tone. The qubit frequency, unfortunately, turned out to be close to the edge of the passband, but we did not observe leakage of the drive tone (Methods and Extended Data Fig. 6) and were able to perform the experiment. The other components between the qubit–resonator and bolometer chips are needed only to individually characterize the qubit–resonator chip (by toggling the double-pole double-throw switch and measuring from the auxiliary readout port) and the bolometer (by applying a pulse from the auxiliary drive port through the directional coupler), and can be removed in future experiments.

The Thermocoax cable in the auxiliary drive line acts as a low-pass filter to prevent high-frequency radiation from leaking in; consequently, we needed to apply roughly 25 dBm higher power at *f*_d_ = 5.5 GHz during the initial bolometer characterization than when driving via the qubit chip.

To probe the bolometer, a continuous microwave tone is first attenuated by 30 dB for the available output power of the source to lie within a suitable range for our experiment. Subsequently, this tone is split into two paths, namely, a reference and a signal. The signal is directed into the cryostat, reflected off of the probe port of the bolometer, and amplified by an HEMT amplifier at 4 K and additional amplifiers at room temperature. Isolators are placed between the HEMT and bolometer to attenuate the thermal noise from the HEMT. Such noise may heat up the bolometer and thus degrade the SNR. However, it is possible to operate the bolometer without isolators on the probe output line, as demonstrated elsewhere^26^, as well as in some of the measurements discussed below. Thermal noise from 4 K over the *κ*_b_ = 7.6 MHz linewidth of the bolometer has a power of *k*_B_*Tκ*_b_ = −123.8 dBm, which is nearly an order of magnitude below the powers of the probe and qubit readout tones. Removing the isolators from the probe output line, thus, seems to be a feasible option.

At room temperature, filters and 3 dB attenuators are placed between the amplifiers to avoid standing waves and possible amplifier saturation. After amplification, the signal is demodulated to an intermediate frequency using a local oscillator, which is detuned from the probe tone by a fixed intermediate frequency of 70.3125 MHz. The reference is demodulated by the local oscillator without passing through the cryostat, and is used as an amplitude and phase reference for the signal. Both intermediate-frequency signals are amplified further and digitized by the NI-5782 module at a sampling rate of 250 megasamples per second and digitally demodulated into in-phase (*I*) and quadrature (*Q*) components. The NI-5782 module also handles the ensemble averaging of the data, where applicable, as well as downsampling by boxcar averaging a variable number of adjacent points. From the time-domain data in the *I*–*Q* plane, we calculate the average signal before the pulse (${\tilde{V}}_{0}$) and the average signal during the pulse ($\tilde{V}$), as determined by *t*_0_ and *t*_RO_. The data are then rotated in the *I*–*Q* plane such that $\tilde{V}-{\tilde{V}}_{0}$ lies on the *I* axis. We define $\bar{V}$ and ${\bar{V}}_{0}$ (discussed in the main text) as the *I* components of $\tilde{V}$ and ${\tilde{V}}_{0}$, respectively, after applying this rotation. For the single-shot experiments, we apply a common rotation across all the shots, with the rotation angle chosen such that the readout fidelity is maximized. Note that the phase of the signal in the *I*–*Q* plane is due to the reflection off of the effective *LC* circuit of the bolometer and is thus completely independent of the phase of the photons emitted by the readout resonator.

The data acquisition and storage is managed by the QCoDeS^45^ data acquisition framework. Data analysis and fitting are carried out using the NumPy^46^, SciPy^47^, xarray^48^ and lmfit^49^ libraries.

### Sample characterization

**Extended Data Fig. 4:**
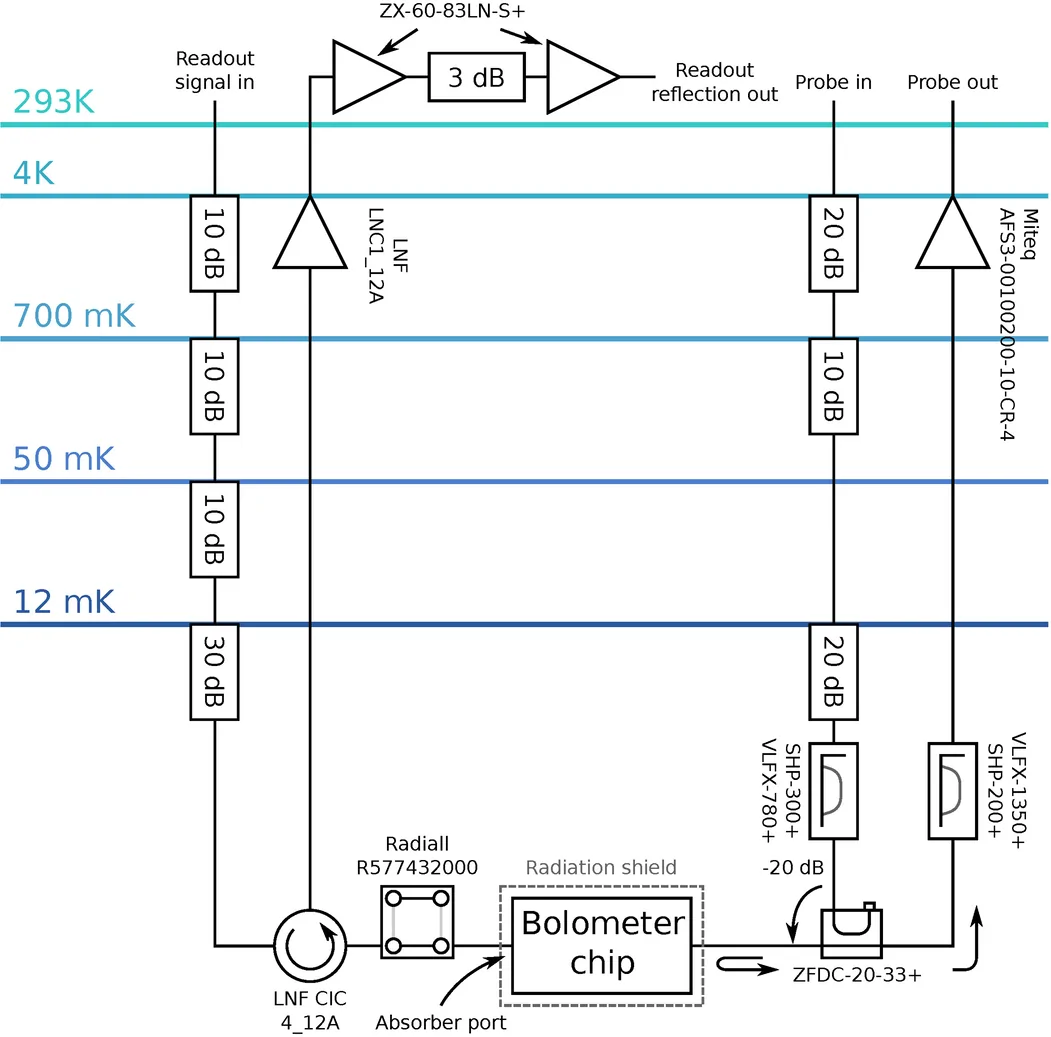
Experimental setup for gain compression measurements. The room-temperature components connected to the probe input and output are as in Extended Data Fig. 1. To characterize the reflection coefficient at the absorber port of the bolometer, we connect an Agilent N9928A vector network analyzer to the readout input and output ports, and for the gain compression measurement we use the same microwave source for the readout input as in Extended Data Fig. 1, without the room-temperature filters. See Extended Data Fig. 1 for the definition of the symbols.

**Extended Data Fig. 5:**
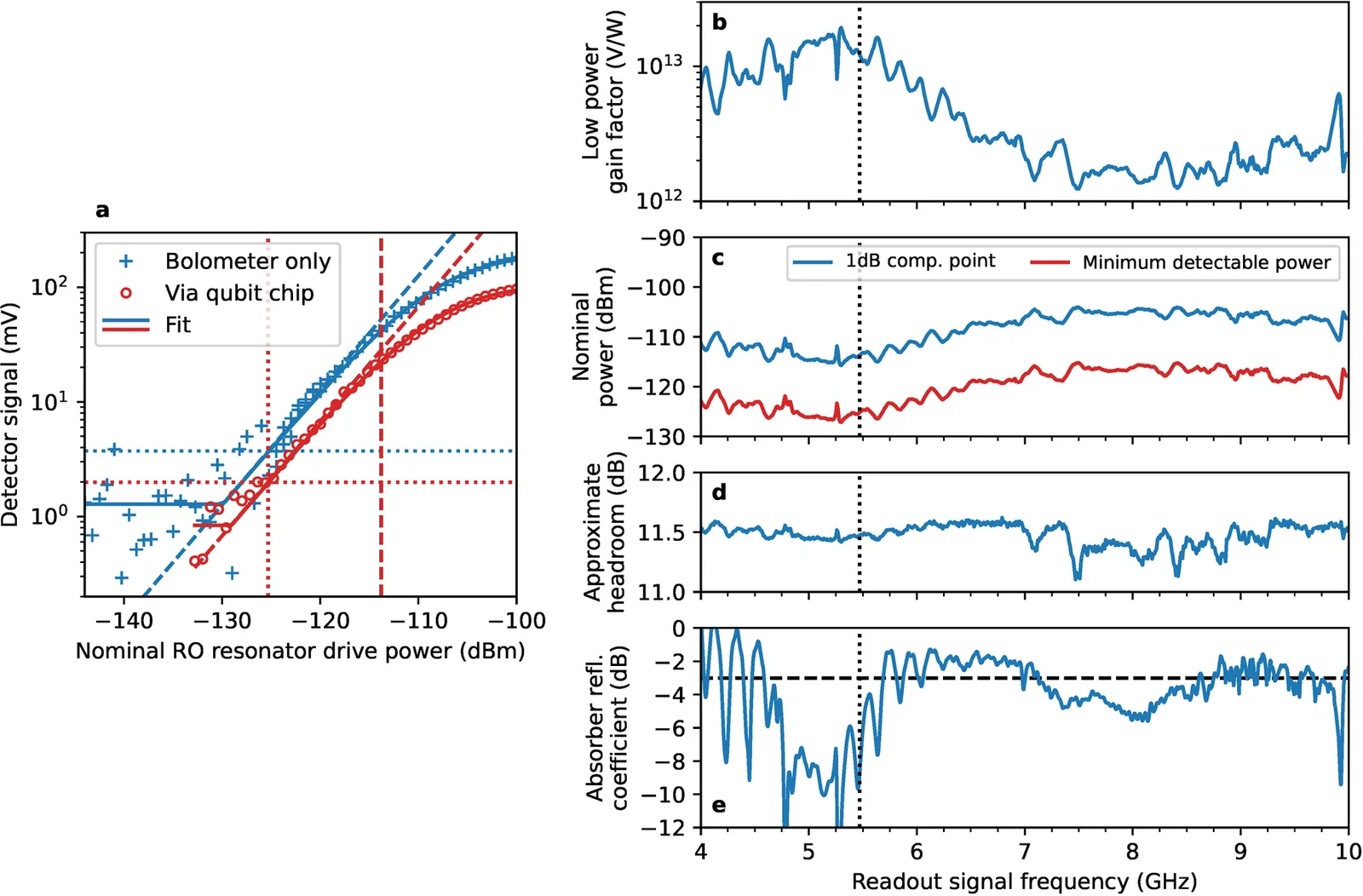
Gain compression and bandwidth. (a) Bolometer detector signal *S* without (blue crosses) and with (red circles) the qubit chip present, as functions of the readout drive power *P*_d_, with *f*_d_ = 5.4 GHz, a 2 ms readout pulse and 16 ensemble averages. The solid lines are fits to a phenomenological model. The horizontal dotted lines indicate the calculated noise floor. The diagonal dashed lines show the signal gain without compression, given by *S* = *G*_0_*P*_d_. The vertical dashed and dotted lines indicate the 1 dB compression point and minimum detectable power, respectively. The compression points for the two datasets are 0.04 dBm apart from each other at −113.8 dBm. The data with the qubit chip present is from the same data set as the data of Fig. 2(d) with *t*_RO_ = 2 ms and *f*_d_ = 5.4 GHz. (b) Low-power gain factor *G*_0_, (c) 1 dB gain compression point (blue) and minimum detectable power (red), (d) headroom defined as the ratio of the gain compression point and the minimum detectable power, and (e) power reflection coefficient ∣Γ_abs_∣^2^ of the bolometer absorber port, as functions of the readout frequency *f*_d_, with no qubit connected to the bolometer. The vertical dotted line indicates the frequency of the readout resonator at 5.47 GHz. The horizontal dashed line in (e) marks -3 dB reflection.

Extended Data Figs. 3–5 show the characterization measurements of the bolometer. Extended Data Fig. 3a shows the signal coming out of the cryostat normalized by the reference, as discussed above. The data are averaged for 1 ms over 16 repetitions. For each pixel in Extended Data Fig. 3b, a 2 ms pulse is applied to the bolometer absorber port via the readout resonator with an off-resonant tone of *f*_d_ = 5.4 GHz and *P*_d_ = −108 dBm. From these data, we extract the time constant *τ*_b_ (Extended Data Fig. 3c) by fitting the time-domain signal to an exponential model.

To characterize the bandwidth of the bolometer, we disconnect the qubit chip and directly connect the readout drive line to the bolometer absorber port without any filters (Extended Data Fig. 4). A circulator and a microwave switch are used in front of the absorber port to be able to measure the reflection coefficient of the absorber, as discussed below. We repeat the measurement shown in Fig. 2d with a readout pulse length of 2 ms and the probe frequency adjusted to 549 MHz. The bolometer resonance frequency shifts owing to the thermal cycling of the cryostat as well as the increased thermal noise from the HEMT since the isolators were removed from the probe output. We vary the readout power *P*_d_ and frequency *f*_d_, and use these data to characterize the gain of the bolometer as follows: for a given readout drive power *P*_d_, the detector signal is given by *S* = *G*(*P*_d_)*P*_d_, where *G* is a gain factor characterizing the power-to-voltage conversion of the bolometer and the total amplification of the amplification chain of the probe signal, including the HEMT and room-temperature amplifiers. We describe the gain suppression of the bolometer using the following simple phenomenological model:

2 $$S({P}_{{{{\rm{d}}}}})=\max \left\{\frac{{G}_{0}}{1+{P}_{{{{\rm{d}}}}}/{P}_{{{{\rm{sat}}}}}}{P}_{{{{\rm{d}}}}},{\bar{V}}_{{{{\rm{noise}}}}}\right\},$$

where *P*_sat_ is the saturation power and *G*_0_ is the linear gain factor at low readout powers, where it coincides with the quasistatic responsivity^28^. Here ${\bar{V}}_{{{{\rm{noise}}}}}$ is the average noise level, calculated as the median of the data across *f*_d_ with *P*_d_ = 0, since with no power applied, the detector signal consists of noise only, independent of *f*_d_. We calculate the noise floor as ${V}_{{{{\rm{NF}}}}}={\bar{V}}_{{{{\rm{noise}}}}}+3{\sigma }_{{f}_{{{{\rm{d}}}}}}(S)$, where ${\sigma }_{{f}_{{{{\rm{d}}}}}}(S)$ is the sample standard deviation of the data across *f*_d_ with *P*_d_ = 0. We define the 1 dB compression point as *P*_1 dB_ = (10^1/10^ − 1)*P*_sat_, which is found by solving *S*(*P*_1 dB_) = 10^−1/10^*G*_0_*P*_1 dB_ from equation (2) with ${\bar{V}}_{{{{\rm{noise}}}}}=0$. The minimum-detectable power *P*_min_ is given by *V*_NF_/*G*_0_, above which *S* from equation (2) is above the noise floor.

Extended Data Fig. 5a shows *S* measured with the qubit chip removed, along with the data for *t*_RO_ = 2 ms and *f*_d_ = 5.4 GHz from Fig. 2d for comparison. For the data shown in Fig. 2d, we calculate ${\bar{V}}_{{{{\rm{noise}}}}}$ and *V*_NF_ from the median and sample standard deviation, respectively, of *S* for the values where *P*_d_ ≤ −128 dBm, since no other data are available. We find excellent agreement between the data and the model of equation (2). Extended Data Fig. 5a also shows the extracted noise floor, *P*_min_ and *P*_1 dB_.

The extracted *G*_0_, *P*_1 dB_ and *P*_min_, as well as the headroom (defined as *P*_1 dB_/*P*_min_), are shown in Extended Data Fig. 5b–d over a broad range of frequencies. Overall, there is around 10 dB of variation in *P*_1 dB_ and *P*_min_, but the headroom is fairly flat and consistently above 11 dB. On the basis of this, the performance of the bolometer can be expected to be similar to that discussed in the main text across a broad range of frequencies, provided that the impedance of the absorber is matched (as discussed below). Note that the gain suppression of the bolometer does not arise from saturation, but rather because the resonance frequency of the bolometer shifts, which results in a smaller change in *S* away from resonance. This also means that *P*_1 dB_, *P*_min_ and the headroom vary with *f*_p_, with the headroom maximized near the resonance.

We further study the impedance matching of the absorber port using the setup shown in Extended Data Fig. 4. A microwave signal from a vector network analyser is sent to the absorber port and measured via a circulator. The obtained *S*_21_ transmission coefficient is normalized by a reference measurement with the microwave switch between the circulator and bolometer connected to a fully reflecting open line. The resulting absorber power reflection coefficient ∣*Γ*_abs_(*f*_d_)∣^2^ is shown in Extended Data Fig. 5e. We find that ∣*Γ*_abs_∣^2^ is independent of the drive power *P*_d_, and ∣*Γ*_abs_∣^2^ ≲ −3 dB ≈ 0.50 for *f*_d_ around 4.6–5.6 GHz and 7.2–8.6 GHz, and ∣*Γ*_abs_∣^2^ ≲ −6 dB ≈ 0.25 for *f*_d_ around 4.7–5.4 GHz. We also observe 10-MHz-wide resonance peaks near 4.79 GHz with ∣*Γ*_abs_∣^2^ < −22 dB ≈ 6 × 10^−3^ and at 5.28 GHz with ∣*Γ*_abs_∣^2^ < −31 dB ≈ 8 × 10^−4^. At *f*_d_ = *f*_r_ ≈ 5.47 GHz, we find ∣*Γ*_abs_∣^2^ = −8.9 dB, which corresponds to a real impedance of *Z*_abs_ = *Z*_0_(1 + *Γ*_abs_)/(1 − *Γ*_abs_) = 106.2 Ω, where *Z*_0_ = 50.0 Ω is the impedance of the coaxial transmission line connected to the bolometer sample holder. We attribute the overall impedance mismatch to reflections from the sample holder, reflections from the bonding wires between the sample holder and chip, and the geometry of the absorber. This suggests that the SNR of the qubit readout can be improved at least by a factor of

3 $$\frac{1-| {\varGamma }_{{{{\rm{abs}}}}}(5.28\,{\rm{GHz}}){| }^{2}}{1-| {\varGamma }_{{{{\rm{abs}}}}}(5.47\,{\rm{GHz}}){| }^{2}}=1.148$$

by placing the qubit and bolometer on the same chip and optimizing the absorber geometry.

**Extended Data Fig. 6:**
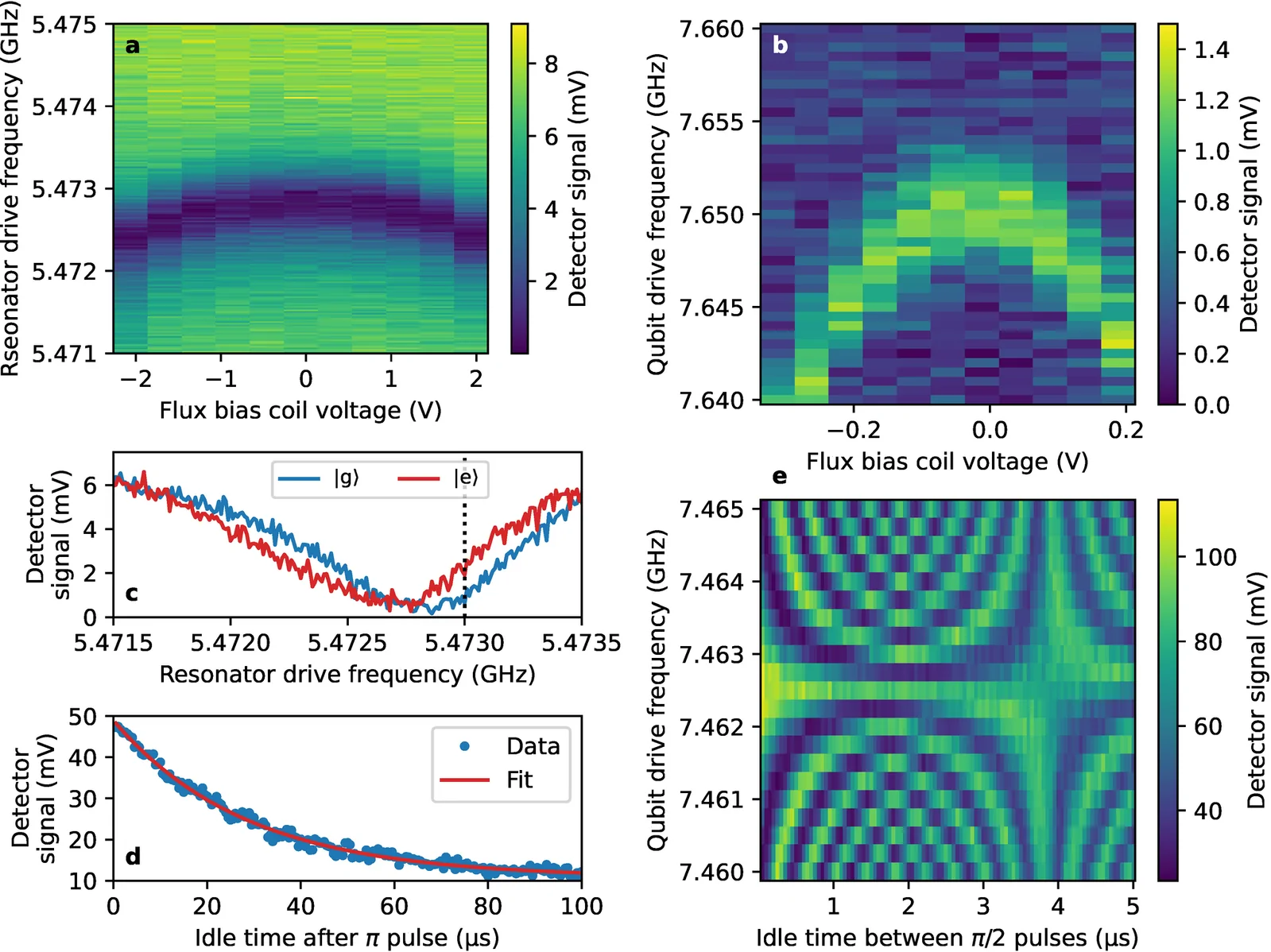
Qubit characterization using bolometer. (a) Detector signal *S* as a function of the bias voltage applied to the flux bias coil and of the frequency of the resonator drive pulse. The resonator frequency, indicated by the minimum detector signal at a given flux value, clearly changes as a function of the flux bias. The data of panel (a) are averaged over 4 repetitions. (b) Detector signal *S* as a function of the flux bias voltage and the frequency of a continuous tone applied to the qubit. The data is averaged over 32 repetitions. The data of panels (a) and (b) are acquired using a readout power of *P*_d_ = − 120.8 dBm and a pulse duration *t*_RO_ = 1 ms. (c) Detector signal *S* as a function of the readout drive frequency *f*_d_, with the qubit prepared in the $\left\vert {{{\rm{g}}}}\right\rangle$ (blue) and $\left\vert {{{\rm{e}}}}\right\rangle$ (red) states. The resonance shifts if the qubit state changes. The vertical dotted line indicates the frequency used for the qubit readout. Here, we have used *P*_d_ = − 109.8 dBm, *t*_RO_ = 10 *μ*s, and averaged over 16384 repetitions. (d) Detector signal *S* as a function of the idling time between a *π* pulse applied to the qubit and the readout pulse, providing the *T*_1_ = 28 *μ*s of the qubit. In these measurements, *P*_d_ = − 112.8 dBm and *t*_RO_ = 20 *μ*s, and the data are averaged over 4096 repetitions. (e) Detector signal as a function of the idle time between the *π*/2 pulses and the qubit drive frequency in a Ramsey experiment which is used to extract the *T*_2_ of the qubit. These data are acquired with *P*_d_ = − 107.8 dBm and *t*_RO_ = 25 *μ*s, averaged over 1024 repetitions.

Extended Data Fig. 6 shows the characterization experiments of the qubit–resonator system using the bolometer. Extended Data Fig. 6a,b shows the single-tone and two-tone spectroscopy, respectively, from which we extract an initial estimate for the resonator and qubit frequencies. Note that in contrast to dispersive readout with voltage amplifiers where the tones may be continuous, we pulse the readout tone in all these experiments. Subsequently, we calibrate the qubit frequency more accurately, as well as the duration of the π pulse (Fig. 2e).

With the π pulse calibrated, we are able to observe the effect of the dispersive shift by measuring the resonator spectrum after preparing the qubit in |*g*〉 and |*e*〉 (Extended Data Fig. 6c). Note that the difference between the locations of the minima in Extended Data Fig. 6c is somewhat smaller than the calculated dispersive shift of *χ*/2π, since the spectrum corresponding to state |*e*〉 is distorted owing to the qubit decaying during readout.

In Fig. 6d, a π pulse is applied to the qubit, and the readout pulse is applied after different delays to extract the *T*_1_ decay time. Extended Data Fig. 6e shows the result of a Ramsey experiment, where the qubit is driven by two π/2 pulses with a varying idle time between them, for different modulation frequencies of the pulse. These data are used to extract the *T*_2_ time, given by the exponentially decaying envelope of the oscillation. The signal level varies in Extended Data Fig. 6, since different values of *P*_d_ and *t*_RO_ were used during the characterization and the optimal values for these parameters were not yet known. In addition, the cryostat was thermally cycled between the acquisition of the data shown in Extended Data Fig. 6a–c and those shown in Extended Data Fig. 6d,e. This is why the qubit frequency has decreased in Extended Data Fig. 6e compared with those in Extended Data Fig. 6b and Fig. 2e.

### Detector signal probability distribution with long averaging time

Following another work^38^, we develop a model for the probability distribution of the detector signal for the qubit nominally prepared in |*e*〉 (with some preparation error) and for the averaging time being comparable with the energy decay time *T*_1_. Let *v*_g/e_(*t*, *t*_d_) be the time-domain signal of the bolometer output when the qubit is in state |*g*〉 or |*e*〉 at the beginning of the readout pulse, that is, *t* = 0. Here *t*_d_ denotes the time at which the qubit instantaneously decays to |*g*〉 if it was initially in |*e*〉 (*v*_g_ is independent of *t*_d_). We assume that no thermal excitation events occur, and that the noise in the signal is Gaussian. Formally, the signal is given by

4 $$\begin{array}{r}{v}_{{{{\rm{g}}}}/{{{\rm{e}}}}}(t,{t}_{{{{\rm{d}}}}}){{{\rm{d}}}}t={u}_{{{{\rm{g}}}}/{{{\rm{e}}}}}(t,{t}_{{{{\rm{d}}}}}){{{\rm{d}}}}t+\sqrt{{P}_{{{{\rm{N}}}}}}\,{{{\rm{d}}}}W(t),\end{array}$$

where *u*_g/e_(*t*, *t*_d_) is the evolution of the output in the absence of noise, *P*_N_ is the noise power spectral density and d*W*(*t*) is the Wiener increment^50^.

To incorporate the bolometer dynamics, we model *u*_g/e_(*t*, *t*_d_) as exponentially approaching some steady-state values *c*_g_ and *c*_e_, for the qubit in state |*g*〉 or |*e*〉, respectively^28^. Namely, we assume that the time evolution is of the form

5 $${u}_{{{{\rm{g}}}}}(t)={c}_{{{{\rm{g}}}}}(1-{{\rm{e}}}^{-t/{\tau }_{{{{\rm{b}}}}}})$$

and

6 $$\begin{array}{r}\begin{array}{rcl}{u}_{{{{\rm{e}}}}}(t,{t}_{{{{\rm{d}}}}})&=&\theta ({t}_{{{{\rm{d}}}}}-t){c}_{{{{\rm{e}}}}}\left(1-{{{{\rm{e}}}}}^{-t/{\tau }_{{{{\rm{b}}}}}}\right)\\ &&+\theta (t-{t}_{{{{\rm{d}}}}})\left[({c}_{{{{\rm{g}}}}}-{c}_{{{{\rm{e}}}}}^{{\prime} })\left(1-{{{{\rm{e}}}}}^{-(t-{t}_{{{{\rm{d}}}}})/{\tau }_{{{{\rm{b}}}}}}\right)+{c}_{{{{\rm{e}}}}}^{{\prime} }\right],\end{array}\end{array}$$

where *θ*(*x*) is the step function and ${c}_{{{{\rm{e}}}}}^{{\prime} }={c}_{{{{\rm{e}}}}}(1-{{{{\rm{e}}}}}^{-{t}_{{{{\rm{d}}}}}/{\tau }_{{{{\rm{b}}}}}})$.

Averaging the bolometer output from *t*_0_ to *t*_RO_ yields the averaged detector signal as

7 $$\begin{array}{rcl}{\bar{V}}_{{{{\rm{g}}}}/{{{\rm{e}}}}}({t}_{{{{\rm{d}}}}})&=&\frac{1}{{t}_{{{{\rm{RO}}}}}-{t}_{0}}\displaystyle\int\nolimits_{{t}_{0}}^{{t}_{{{{\rm{RO}}}}}}{v}_{{{{\rm{g}}}}/{{{\rm{e}}}}}(t,{t}_{{{{\rm{d}}}}}){{{\rm{d}}}}t\\ &=&{\bar{U}}_{{{{\rm{g}}}}/{{{\rm{e}}}}}({t}_{{{{\rm{d}}}}})+X\,[0,{\sigma }^{2}],\end{array}$$

where ${\bar{U}}_{{{{\rm{g}}}}/{{{\rm{e}}}}}({t}_{{{{\rm{d}}}}})\equiv {({t}_{{{{\rm{RO}}}}}-{t}_{0})}^{-1}\int\nolimits_{{t}_{0}}^{{t}_{{{{\rm{RO}}}}}}{u}_{{{{\rm{g}}}}/{{{\rm{e}}}}}(t,{t}_{{{{\rm{d}}}}})\,{{{\rm{d}}}}t$ and *X*[0, *σ*^2^] is a normally distributed random variable with mean 0 and standard deviation $\sigma =\sqrt{{P}_{{{{\rm{N}}}}}/({t}_{{{{\rm{RO}}}}}-{t}_{0})}$. Above, we have neglected the constant ${\bar{V}}_{0}$, which simply shifts ${\bar{V}}_{{{{\rm{g}}}}/{{{\rm{e}}}}}$. With a fixed *t*_d_, the probability of obtaining a given detector voltage *V*, thus, obeys the following probability distribution:

8 $$\begin{array}{r}{P}_{{{{\rm{g}}}}/{{{\rm{e}}}}}\left(V\,| {t}_{{{{\rm{d}}}}}\right)=\frac{1}{\sqrt{2\pi {\sigma }^{2}}}\exp \left\{-\frac{{\left[V-{\bar{U}}_{{{{\rm{g}}}}/{{{\rm{e}}}}}({t}_{{{{\rm{d}}}}})\right]}^{2}}{2{\sigma }^{2}}\right\},\end{array}$$

which is a Gaussian centred around ${\bar{U}}_{{{{\rm{g}}}}/{{{\rm{e}}}}}({t}_{{{{\rm{d}}}}})$ having standard deviation *σ* as defined above.

If the qubit is in state |*g*〉 at *t* = 0, the probability distribution *P*_g_(*V*) is simply a Gaussian centred around the constant value ${\bar{U}}_{{{{\rm{g}}}}}$. When the qubit is initially in |*e*〉, the probability distribution is obtained by calculating the average of *P*_e_(*V*∣*t*_d_) for all the possible realizations of the qubit decay time *t*_d_ weighted by the probability density *P*(*t*_d_) of the decay occurring at *t*_d_:

9 $$\begin{array}{rc}{P}_{{{{\rm{e}}}}}(V\,)&=\displaystyle\int\nolimits_{0}^{\infty }P(V\,| {t}_{{{{\rm{d}}}}})P({t}_{{{{\rm{d}}}}})\,{{{\rm{d}}}}{t}_{{{{\rm{d}}}}},\end{array}$$

where *P*(*t*_d_) is exponentially distributed: $P({t}_{{{{\rm{d}}}}})={{{{\rm{e}}}}}^{-{t}_{{{{\rm{d}}}}}/{T}_{1}}/{T}_{1}$. Thus, the total probability distribution for the qubit nominally prepared in |*e*〉 is the following weighted sum of these distributions:

10 $$\begin{array}{r}{P}_{{{{\rm{e}}}}}^{{{{\rm{tot}}}}}(V\,)={P}_{{{{{x}}}}}{P}_{{{{\rm{g}}}}}(V\,)+(1-{P}_{{{{{x}}}}}){P}_{{{{\rm{e}}}}}(V\,),\end{array}$$

where *P*_*x*_ is the probability that the qubit was actually in state |*g*〉 at *t* = 0. Here *P*_*x*_ includes state preparation errors, as well as the *T*_1_ decay of the qubit during the short delay between the application of the π pulse and the start of the readout pulse.

In the case where *u*_g/e_(*t*, *t*_d_) is like step functions, equations (9) and (10) have analytical expressions^38^. However, we do not obtain an analytical expression for *P*_e_(*V*) given the temporal evolution arising from equation (6), and hence, we numerically calculate the integral of equation (9). Scaling this by the bin width used in Fig. 3b produces the prediction for the distribution, with *c*_g_, *c*_e_, *T*_1_, *σ* and *P*_*x*_ as the fitting parameters. For the data shown in Fig. 3b, using the separately determined value of *τ*_b_ = 9.4 μs, we find *c*_g_ = 24.7 mV, *c*_e_ = 182.0 mV, *T*_1_ = 25.8 μs, *σ* = 17.4 mV and *P*_*x*_ = 0.20.

The readout fidelity is given by

11 $$F=1-\int\nolimits_{-\infty }^{{V}_{{{{\rm{th}}}}}}{P}_{{{{\rm{e}}}}}^{{{{\rm{tot}}}}}(V\,)\,{{{\rm{d}}}}V-\int\nolimits_{{V}_{{{{\rm{th}}}}}}^{\infty }{P}_{{{{\rm{g}}}}}(V\,)\,{{{\rm{d}}}}V,$$

where *V*_th_ is the threshold value for assigning the measurement outcome to |*g*〉 or |*e*〉, and we again assume that no thermal excitations occur.

If we further assume that *P*_*x*_ = 0 and that the qubit never relaxes, ${P}_{{{{\rm{e}}}}}^{{{{\rm{tot}}}}}(V\,)$ reduces to a Gaussian distribution centred around ${\bar{U}}_{{{{\rm{e}}}}}^{\infty }={\bar{U}}_{{{{\rm{e}}}}}({t}_{{{{\rm{d}}}}}\to \infty )$, which can be calculated by dropping the second term in equation (6). In this case, the readout fidelity is maximized with ${V}_{{{{\rm{th}}}}}=({\bar{U}}_{{{{\rm{e}}}}}^{\infty }+{\bar{U}}_{{{{\rm{g}}}}})/2$, which yields

12 $${F}^{\infty }={{{\rm{erf}}}}\left(\frac{{\bar{U}}_{{{{\rm{e}}}}}^{\infty }-{\bar{U}}_{{{{\rm{g}}}}}}{2\sqrt{2{\sigma }^{2}}}\right),$$

where erf(*x*) is the error function. Using the values for *c*_g_, *c*_e_, *τ*_b_ and *σ* extracted from the fitting, and fixing *t*_RO_ = 13.9 μs, we calculate ${\bar{U}}_{{{{\rm{g}}}}}$ and ${\bar{U}}_{{{{\rm{e}}}}}^{\infty }$ as functions of *t*_0_. After inserting these into equation (12), we maximize *F*^∞^ with respect to *t*_0_ and find *F*^∞^ ≈ 0.927 with *t*_0_ = 3.19 μs.

### Estimate of parameters needed for 99.9% fidelity

Here we quantitatively discuss how to achieve 99.9% fidelity with bolometric readout following improvements to the experiment proposed in the main text. To be able to make a fair comparison between the performance of different bolometers presented in the literature^28,29^, we carry out this analysis by assuming that we operate in the calorimetric mode. Namely, we interpret the distributions shown in Fig. 3b to represent a measurement of the energy of the absorbed pulses. In such an experiment, the signal is directly proportional to the energy packet arriving at the bolometer, whereas the width of the distribution is independent of the energy.

Let us first assume that the length of the readout pulse is reduced to 200 ns without changing the readout power. This means that the bolometer-absorbed energy, and hence the SNR (Fig. 3b), is reduced by a factor of 70. By changing the metallic bolometer to a graphene bolometer, the energy resolution has been observed to increase by a factor of 13 (ref. ^29^). Thus, to obtain 99.9% fidelity, one needs to increase the amount of energy absorbed by the graphene bolometer in 200 ns by a factor of *A*_99.9%_ ≈ 1.8 × 70/13 ≈ 10, where the factor of 1.8 arises from the fact that to reduce the overlap infidelity of the data shown in Fig. 3b from 7.0% to 0.1%, one needs a factor of 1.8 improvement in the SNR in equation (12).

Below, we aim to show that by making different improvements to the measurement scheme, we can arrive at several factors *A*_*α*_ of increment to the energy absorbed by the bolometer; taking all these factors into account, it is possible to exceed the required SNR, that is, *A*_99.9%_ = 10 < *∏*_*α*_*A*_*α*_. Since we have assumed a 200 ns readout time, having a qubit *T*_1_ of the order of 100 μs is sufficient to reach 99.9% readout fidelity. Note that the bolometer is a power sensor, and thus, the SNR is directly proportional to the absorbed power for the low-enough powers considered here^29^ (Fig. 2d).

First, switching to a transmission-type setup, where all the readout photons are directed into the bolometer instead of half of them escaping through the input, increases the power incident on the bolometer by a factor of *A*_t_ = 2. Second, removing additional components between the qubit and bolometer chips reduces the losses by approximately *A*_c_ ≈ 1 dB ≈ 1.250, and improving the impedance matching of the absorber increases the SNR by a factor of *A*_*Z*_ = 1.148 according to equation (3). Third, by optimizing the dispersive shift *χ* for photodetection-type readout, we expect that the photon number occupying the resonator during readout, and thus the incident power, can be increased by a factor of *A*_*χ*_ = 2 (ref. ^32^). Fourth, switching the qubit–resonator system to a configuration where the resonance frequency of the resonator lies above the qubit frequency (keeping the detuning ∣*f*_r,g_ − *f*_q_∣ constant) allows driving with a larger number of photons^36^, which we estimate to be at least *A*_a_ = 1.5 times greater than with our current parameters. Fifth, increasing the admissible drive power by doubling the resonator drive frequency to 10 GHz may further double the photon energy *hf*_r,g_, introducing a factor of *A*_2*f*_ = 2. In total, the improvement in SNR is given by

13 $${A}_{{{{\rm{t}}}}}{A}_{{{{\rm{c}}}}}{A}_{Z}{A}_{{{{\rm{a}}}}}{A}_{2f}{A}_{\chi }\approx 17.3 > {A}_{99.9 \% },$$

and thus, a high fidelity is feasibly achievable.

### Multiplexed qubit readout using bolometers

**Extended Data Fig. 7:**
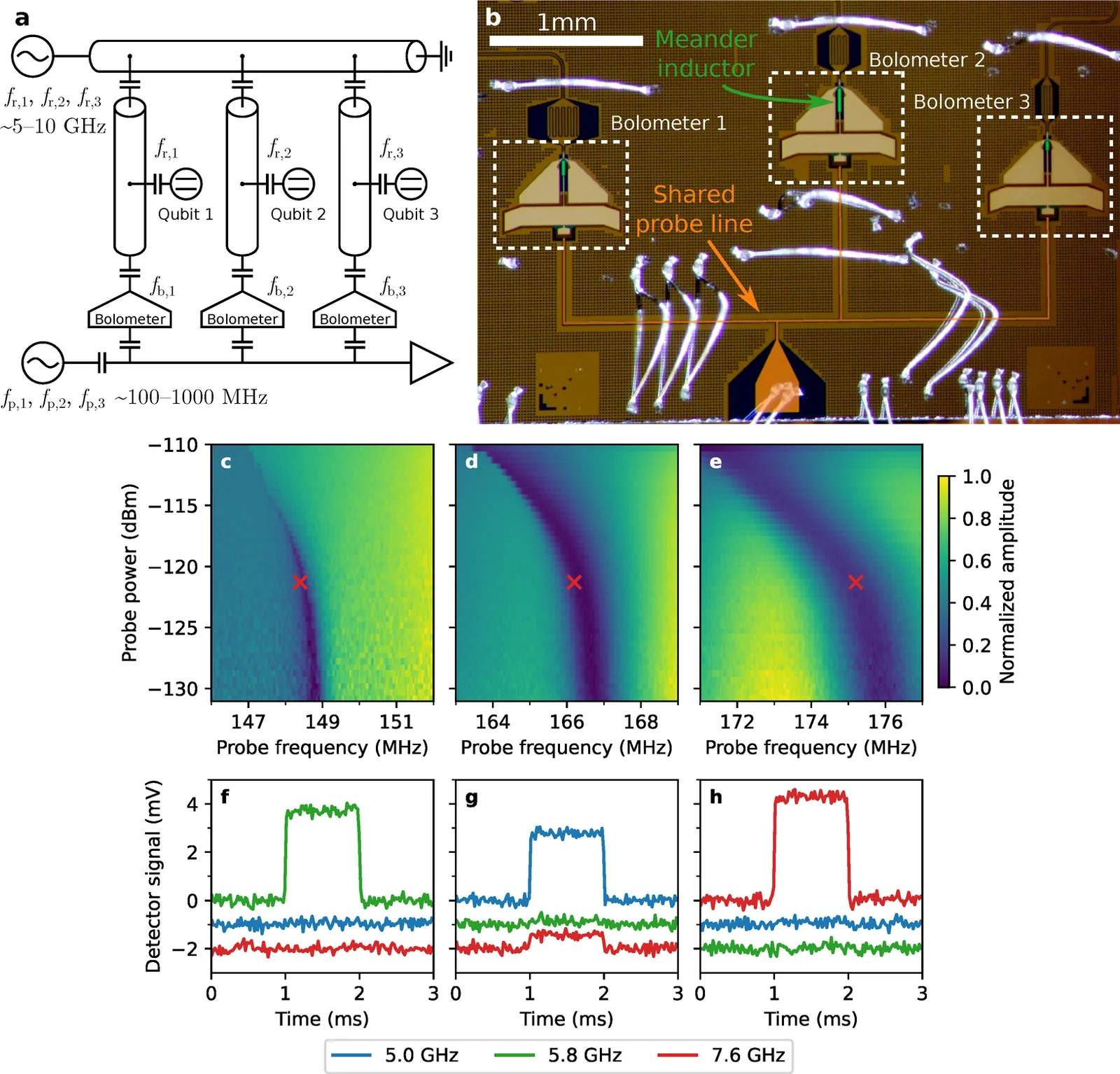
Multiplexed qubit readout using bolometers. (a) Illustration of the proposed scheme for multiplexed qubit readout using bolometers. Readout resonators with resonance frequencies *f*_r,*i*_ in the gigahertz range are driven via a shared feedline and coupled to individual bolometers with resonance frequencies *f*_b,*i*_ in the sub-gigahertz range. The bolometers are probed with separate probe frequencies *f*_p,*i*_ ≈ *f*_b,*i*_ via a shared probe line. (b) False-color optical micrograph of a chip with three bolometers sharing a probe line (orange). The meandering inductors (green) are used to tune the frequency of the *LC* circuits. (c)–(e) Normalized transmission amplitudes near the resonance frequencies of the three bolometers as functions of the probe frequency and probe power. (f)–(h) Measured detector signals as functions of time with 1 ms pulses applied to the absorber ports of the different bolometers at 1 ms. Each bolometer is driven via input passband filters (not shown) at their absorber port by one pulse at a time with frequency 5.0 GHz (blue), 5.8 GHz (green), or 7.6 GHz (red). For each bolometer, the signal is large only for one of the drive frequencies, indicating that the cross-talk is small. The signals corresponding to the stopband drive frequencies are offset by − 1 and −2 mV for clarity. The red cross in each of the panels (b)–(d) mark the probe parameters that were used to acquire the data in each of the panels (e)–(g), respectively.

Let us discuss the applicability of bolometer-based qubit readout for quantum processors consisting of multiple qubits. Although a bolometer cannot natively distinguish the frequency of incident photons, frequency-multiplexed qubit readout is feasible by coupling individual bolometers to the readout resonators of each qubit (Extended Data Fig. 7a). Here it is possible to multiplex the bolometer probe signals in the frequency domain using a shared probe line. Furthermore, this way of multiplexing provides relief to the available headroom in the total probe power since probing at the resonance frequency of one bolometer does not saturate the other bolometers. In three-dimensional integrated quantum processors^40^, these individual bolometers may be placed on the wiring layer below the qubits owing to the small footprint of the bolometers.

To take the first steps in demonstrating the feasibility of using bolometers for multiplexed qubit readout, we have fabricated a chip with three bolometers (Extended Data Fig. 7b). Each bolometer has a separate readout input port, which may be connected to qubit readout resonators. The design of bolometers is similar to the sample discussed in the main text, apart from the addition of meander-line inductors in series with the nanowire to control the designed resonance frequencies of the bolometer *LC* circuits. Extended Data Fig. 7c–h shows the preliminary characterization results from this three-bolometer device. We find three distinct resonances (Extended Data Fig. 7c–e), which shift down in frequency with increasing probe power, indicating that they correspond to bolometers that heat up due to electrothermal feedback. We are able to clearly observe microwave signals between 5.0 and 7.6 GHz sent to the absorbers of each bolometer through separate input lines without major cross-talk (Extended Data Fig. 7f–h). These preliminary results on multiplexed bolometeric readout pave the way for future experiments where multiple qubits are read by their dedicated bolometer.

## Data Availability

The data that support the findings of this study are available via Zenodo at https://doi.org/10.5281/zenodo.7773980 (ref. ^51^).

## References

[1] DiVincenzo, D. P. The physical implementation of quantum computation. *Fortschr. Phys.* **48**, 771–783 (2000).

[2] Nielsen, M. A. & Chuang, I. L. *Quantum Computation and Quantum Information: 10th Anniversary Edition* (Cambridge Univ. Press, 2011).

[3] Shor, P. W. Scheme for reducing decoherence in quantum computer memory. *Phys. Rev. A* **52**, R2493–R2496 (1995). 9912632 10.1103/physreva.52.r2493

[4] Preskill, J. Fault-tolerant quantum computation. Preprint at https://arxiv.org/abs/quant-ph/9712048 (1997).

[5] Terhal, B. M. Quantum error correction for quantum memories. *Rev. Mod. Phys.* **87**, 307–346 (2015).

[6] Blais, A., Huang, R.-S., Wallraff, A., Girvin, S. M. & Schoelkopf, R. J. Cavity quantum electrodynamics for superconducting electrical circuits: an architecture for quantum computation. *Phys. Rev. A* **69**, 062320 (2004).

[7] Barends, R. et al. Logic gates at the surface code threshold: superconducting qubits poised for fault-tolerant quantum computing. *Nature* **508**, 500–503 (2014). 24759412 10.1038/nature13171

[8] Kjaergaard, M. et al. Superconducting qubits: current state of play. *Annu. Rev. Condens. Matter Phys.* **11**, 369–395 (2020).

[9] Zhao, Y. et al. Realization of an error-correcting surface code with superconducting qubits. *Phys. Rev. Lett.* **129**, 030501 (2022). 35905349 10.1103/PhysRevLett.129.030501

[10] Krinner, S. et al. Realizing repeated quantum error correction in a distance-three surface code. *Nature* **605**, 669–674 (2022). 35614249 10.1038/s41586-022-04566-8

[11] Acharya, R. et al. Suppressing quantum errors by scaling a surface code logical qubit. *Nature* **614**, 676–681 (2023). 36813892 10.1038/s41586-022-05434-1 PMC9946823

[12] Koch, J. et al. Charge-insensitive qubit design derived from the Cooper pair box. *Phys. Rev. A* **76**, 042319 (2007).

[13] Blais, A., Grimsmo, A. L., Girvin, S. M. & Wallraff, A. Circuit quantum electrodynamics. *Rev. Mod. Phys.* **93**, 025005 (2021).

[14] Walter, T. et al. Rapid high-fidelity single-shot dispersive readout of superconducting qubits. *Phys. Rev. Appl.* **7**, 054020 (2017).

[15] Sunada, Y. et al. Fast readout and reset of a superconducting qubit coupled to a resonator with an intrinsic Purcell filter. *Phys. Rev. Appl.* **17**, 044016 (2022).

[16] Heinsoo, J. et al. Rapid high-fidelity multiplexed readout of superconducting qubits. *Phys. Rev. Appl.* **10**, 034040 (2018).

[17] Aumentado, J. Superconducting parametric amplifiers: the state of the art in Josephson parametric amplifiers. *IEEE Microw. Magazine* **21**, 45–59 (2020).

[18] Esposito, M., Ranadive, A., Planat, L. & Roch, N. Perspective on traveling wave microwave parametric amplifiers. *Appl. Phys. Lett.* **119**, 120501 (2021).

[19] Clerk, A. A., Devoret, M. H., Girvin, S. M., Marquardt, F. & Schoelkopf, R. J. Introduction to quantum noise, measurement, and amplification. *Rev. Mod. Phys.* **82**, 1155–1208 (2010).

[20] Bergeal, N. et al. Phase-preserving amplification near the quantum limit with a Josephson ring modulator. *Nature* **465**, 64–68 (2010). 20445625 10.1038/nature09035

[21] Macklin, C. et al. A near–quantum-limited Josephson traveling-wave parametric amplifier. *Science* **350**, 307–310 (2015). 26338795 10.1126/science.aaa8525

[22] Opremcak, A. et al. High-fidelity measurement of a superconducting qubit using an on-chip microwave photon counter. *Phys. Rev. X* **11**, 011027 (2021).

[23] Chen, L. et al. Transmon qubit readout fidelity at the threshold for quantum error correction without a quantum-limited amplifier. *NPJ Quantum Inf.* **9**, 26 (2023).

[24] Lecocq, F. et al. Control and readout of a superconducting qubit using a photonic link. *Nature* **591**, 575–579 (2021). 33762768 10.1038/s41586-021-03268-x

[25] Delaney, R. D. et al. Superconducting-qubit readout via low-backaction electro-optic transduction. *Nature* **606**, 489–493 (2022). 35705821 10.1038/s41586-022-04720-2

[26] Govenius, J. et al. Microwave nanobolometer based on proximity Josephson junctions. *Phys. Rev. B* **90**, 064505 (2014).

[27] Govenius, J., Lake, R. E., Tan, K. Y. & Möttönen, M. Detection of zeptojoule microwave pulses using electrothermal feedback in proximity-induced Josephson junctions. *Phys. Rev. Lett.* **117**, 030802 (2016). 27472107 10.1103/PhysRevLett.117.030802

[28] Kokkoniemi, R. et al. Nanobolometer with ultralow noise equivalent power. *Commun. Phys.* **2**, 124 (2019). 10.1038/s42005-019-0225-6 PMC11938398 40144809

[29] Kokkoniemi, R. et al. Bolometer operating at the threshold for circuit quantum electrodynamics. *Nature* **586**, 47–51 (2020). 32999484 10.1038/s41586-020-2753-3

[30] Girard, J.-P. et al. Cryogenic sensor enabling broad-band and traceable power measurements. *Rev. Sci. Instrum.* **94**, 054710 (2023). 37227193 10.1063/5.0143761

[31] Barends, R. et al. Coherent Josephson qubit suitable for scalable quantum integrated circuits. *Phys. Rev. Lett.* **111**, 080502 (2013). 10.1103/PhysRevLett.111.080502 24010421

[32] Nesterov, K. N., Pechenezhskiy, I. V. & Vavilov, M. G. Counting statistics of microwave photons in circuit QED. *Phys. Rev. A* **101**, 052321 (2020).

[33] Gambetta, J. et al. Quantum trajectory approach to circuit QED: quantum jumps and the Zeno effect. *Phys. Rev. A* **77**, 012112 (2008).

[34] Chen, Z. *Metrology of Quantum Control and Measurement in Superconducting Qubits*. PhD thesis, Univ. California Santa Barbara (2018).

[35] Khezri, M. et al. Measurement-induced state transitions in a superconducting qubit: within the rotating wave approximation. *Phys. Rev. Appl.* **20**, 054008 (2023). 10.1103/PhysRevLett.117.190503 27858439

[36] Cohen, J., Petrescu, A., Shillito, R. & Blais, A. Reminiscence of classical chaos in driven transmons. *PRX Quantum* **4**, 020312 (2023).

[37] Scott, D. W. On optimal and data-based histograms. *Biometrika* **66**, 605–610 (1979).

[38] Gambetta, J., Braff, W. A., Wallraff, A., Girvin, S. M. & Schoelkopf, R. J. Protocols for optimal readout of qubits using a continuous quantum nondemolition measurement. *Phys. Rev. A* **76**, 012325 (2007).

[39] McKitterick, C. B., Prober, D. E. & Karasik, B. S. Performance of graphene thermal photon detectors. *J. Appl. Phys.* **113**, 044512 (2013).

[40] Kosen, S. et al. Building blocks of a flip-chip integrated superconducting quantum processor. *Quantum Sci. Technol.* **7**, 035018 (2022).

[41] Touzard, S. et al. Gated conditional displacement readout of superconducting qubits. *Phys. Rev. Lett.* **122**, 080502 (2019). 30932609 10.1103/PhysRevLett.122.080502

[42] Ikonen, J. et al. Qubit measurement by multichannel driving. *Phys. Rev. Lett.* **122**, 080503 (2019). 10.1103/PhysRevLett.122.080503 30932559

[43] Lienhard, B. et al. Deep-neural-network discrimination of multiplexed superconducting-qubit states. *Phys. Rev. Appl.* **17**, 014024 (2022).

[44] Siddiqi, I. et al. RF-driven Josephson bifurcation amplifier for quantum measurement. *Phys. Rev. Lett.* **93**, 207002 (2004). 15600958 10.1103/PhysRevLett.93.207002

[45] Nielsen, J. H. et al. QCoDeS/Qcodes: 0.34.0 - June 2022 (2022-06-13). *Zenodo*10.5281/zenodo.6637581 (2022).

[46] Harris, C. R. et al. Array programming with NumPy. *Nature* **585**, 357–362 (2020). 32939066 10.1038/s41586-020-2649-2 PMC7759461

[47] Virtanen, P. et al. SciPy 1.0: fundamental algorithms for scientific computing in Python. *Nat. Methods* **17**, 261–272 (2020). 10.1038/s41592-019-0686-2 PMC7056644 32015543

[48] Hoyer, S. & Joseph, H. xarray: N-D labeled arrays and datasets in Python. *J. Open Res. Software* **5**, 10 (2017).

[49] Newville, M. et al. lmfit/lmfit-py: 1.1.0. *Zenodo*10.5281/zenodo.3588521 (2019).

[50] Jacobs, K. *Stochastic Processes for Physicists: Understanding Noisy Systems* (Cambridge Univ. Press, 2010).

[51] Gunyho, A. M. et al. Data for “Single-shot readout of a superconducting qubit using a thermal detector”. *Zenodo*10.5281/zenodo.10000511 (2023).

